# High-level production and purification in a functional state of an extrasynaptic gamma-aminobutyric acid type A receptor containing α4β3δ subunits

**DOI:** 10.1371/journal.pone.0191583

**Published:** 2018-01-19

**Authors:** Xiaojuan Zhou, Rooma Desai, Yinghui Zhang, Wojciech J. Stec, Keith W. Miller, Youssef Jounaidi

**Affiliations:** Department of Anesthesia, Critical Care and Pain Medicine, Massachusetts General Hospital, Harvard Medical School, Boston, Massachusetts, United States of America; McLean Hospital/ Harvard Medical School, UNITED STATES

## Abstract

The inhibitory γ-aminobutyric acid type A receptors are implicated in numerous physiological processes, including cognition and inhibition of neurotransmission, rendering them important molecular targets for many classes of drugs. Functionally, the entire GABA_A_R family of receptors can be subdivided into phasic, fast acting synaptic receptors, composed of α-, β- and γ-subunits, and tonic extrasynaptic receptors, many of which contain the δ-subunit in addition to α- and β-subunits. Whereas the subunit arrangement of the former group is agreed upon, that of the αβδ GABA_A_Rs remains unresolved by electrophysiological and pharmacological research. To resolve such issues will require biophysical techniques that demand quantities of receptor that have been previously unavailable. Therefore, we have engineered a stable cell line with tetracycline inducible expression of human α4-, β3- and N-terminally Flag-tagged δ-subunits. This cell line achieved a specific activity between 15 and 20 pmol [^3^H]muscimol sites/mg of membrane protein, making it possible to obtain 1 nmole of purified α4β3δ GABA_A_R from sixty 15–cm culture dishes. When induced, these cells exhibited agonist–induced currents with characteristics comparable to those previously reported for this receptor and a pharmacology that included strong modulation by etomidate and the δ-subunit-specific ligand, DS2. Immunoaffinity purification and reconstitution in CHAPS/asolectin micelles resulted in the retention of equilibrium allosteric interactions between the separate agonist, anesthetic and DS2 sites. Moreover, all three subunits retained glycosylation. The establishment of this well–characterized cell line will allow molecular level studies of tonic receptors to be undertaken.

## Introduction

GABA_A_Rs, which are members of the Cys-loop pentameric ligand-gated ion channel superfamily, are the main inhibitory neuroreceptors in the brain and the target of many drugs and endogenous ligands. Most naturally occurring GABA_A_Rs are heteropentamers made up from the 19 known highly homologous subunits (for a review see Olsen and Sieghart (1)). The functional properties of GABA_A_Rs depend on their subunit composition. Two important classes of GABA_A_R in the brain are the synaptic receptors that all contain αβγ subunits and the extra–synaptic receptors that often contain αβδ subunits [1]. These receptors mediate phasic and tonic currents respectively (reviewed in [2, 3]). The agonist GABA activates tonic receptors at lower concentrations and with lower efficacy than phasic receptors. The tonic receptors are of great significance, being thought to be involved in sleep, stress, psychiatric disorders, epilepsy, learning, memory and neuroprotection [3].

Because positive allosteric effectors act in interfaces between subunits, the variable composition and arrangement of each GABA_A_R’s subunits provides opportunities for the development of drugs that bind selectively to different receptor subtypes [4, 5]. For example, the synaptic receptors are targeted by benzodiazepines that bind between α- and γ-subunits in the extracellular domain, whereas the general anesthetic etomidate acts on tonic and phasic receptors by binding between β (except β1) and α1–6 subunits in the transmembrane domain. With the exception of DS2 and its analogs, which selectively modulate tonic δ-subunit containing GABA_A_Rs, no other drugs specific to those receptors have been developed [6].

Whilst phasic receptors are commonly agreed to be arranged β–α–β–α–γ, there is considerable uncertainty surrounding the stoichiometry and arrangement of the α-, β- and δ-subunits in tonic receptors. Electrophysiological studies in oocytes suggest that several arrangements are possible depending on the ratio of constructs transfected [7, 8]. However, the properties of these receptors depend on the expression system, and it has been reported that expression in HEK cells leads to less variability with the most likely arrangement being β2-α4-β2-α4-δ [9]. On the other hand, even in HEK cells the subunit stoichiometry depends on the ratio of the constructs used in transfection; titration of construct ratios in α1β2δ receptors in HEK cells led to the conclusion that several subunit arrangements are possible [10]. In a thorough quantitative Western blotting study of α4β2δ receptors in which subunit transfection ratios were varied, it was concluded that the amount of the δ-construct transfected correlated with the δ-subunit incorporated into the pentamer at the expense of the β2-subunit [11].

Given the above issues, we sought to develop a stable cell line that expressed large quantities of tonic α4β3δ GABA_A_Rs. The advantages of such a high yielding cell line would include the ability to study gating in receptors with low open probability (P_open_) and to undertake biophysical and structural studies in purified, reconstituted receptors. Here, extending our previous work on α1β3 and synaptic α1β3γ2 GABA_A_Rs [12, 13], we report the high-level expression, electrophysiological characterization, purification, and functional reconstitution into CHAPS/asolectin micelles of human extrasynaptic α4β3δ GABA_A_Rs using an inducible HEK293-TetR cell line [14].

## Materials and methods

### Cloning of α_4_ and δ subunits

A human brain cDNA library (Clontech, Mountain View, CA) was used to clone the cDNAs of GABA_A_ receptor α4- and δ-subunits. A 1665 bp cDNA fragment corresponding to 984–2650 base pairs of α4-subunit (GenBank Accession # NM_000809) and a 1359 bp cDNA corresponding to 96–1454 base pairs of δ-subunit (GenBank Accession # NM_000815) were amplified by PCR using DNA polymerase *Pfu* ultra (Agilent Technologies, Santa Clara, CA). The sequences of PCR primers used are specified in S1 Table. The human β3 (NM_021912.3) cDNA was cloned previously [12]. The cDNAs of α4, β3, and δ-subunits were sub-cloned into different tetracycline-inducible, mammalian expression vectors that conferred resistance to different drugs. The α4-subunit was sub-cloned into pCDNA4/TO (zeocin; Life Technologies, Grand Island, NY), the β3-subunit into pCDNA3.1/TO (hygromycin; Life Technologies) and the δ-subunit into pACMV/TetO (G418/geneticin, Life Technologies). The estimated molecular weight of each genetic construct is 4.43 x 10^6^ g/mol for α4-subunit, 4.59 x 10^6^ g/mol for β3-subunit and 5.86 x 10^6^ g/mol for δ-subunit. Sanger DNA sequencing at MGH DNA core was used to confirm the DNA sequences of all three GABA_A_R subunits.

### *Pfu* Mutagenesis to insert Flag-tag in human GABA_A_R δ-subunit

SignalP 4.1 server (http://www.cbs.dtu.dk/services/SignalP) was used to predict the signal sequence in the α4- and δ-subunit protein sequences [15]. A Flag-tag, DYKDDDDK, was inserted between amino acid 4 and 5 of the predicted mature α4-subunit and amino acids 5 and 6 of the δ-subunit protein by Pfu mutagenesis using DNA polymerase *Pfu* ultra (Agilent Technologies), as outlined in the S1 Text. The respective δ-subunit-pACMV/TetO or α4-subunit-pCDNA4/TO plasmids generated were used as reaction templates. The sequence of the primers used for mutagenesis is specified in S1 Table. Sanger DNA sequencing at MGH DNA core facility confirmed insertion of the Flag-tag at desired locations.

### Transfection protocol

HEK293-TetR cells [14, 16] expressing the tetracycline repressor protein (TetR) were cultured in DMEM/F12 (Life Technologies, Grand Island, NY) supplemented with 10% fetal bovine serum (Atlanta Biologicals, Flowery Branch, GA), 5 μg/ml Blasticidin (Lifesciences), 0.2% penicillin/streptomycin (Life Technologies) at 37°C with 5% CO_2_. Cells seeded at a density of 250,000 cells/well in a 6-well plate were allowed to settle for 24h and transfected with a total of 4 μg DNA using Lipofectamine 2000 (Life Technologies), according to manufacturer instructions. Cells expressing N-Flag-α_4_β_3_ were transfected with DNA constructs at the weight ratio of 2:1 (α4:β3, respectively), whilst α_4_β_3_N-Flag-δ receptor was prepared with 2:1:0.25 (α4:β3:δ, respectively). To select for drug resistant cells, after 48 hours, the growth medium was supplemented with 250 μg/ml zeocin and 50 μg/ml hygromycin for the N-Flag-α4β3 expressing line and additionally with 200 μg/ml G418 for the α4β3N-Flag-δ expressing line for the selection of drug resistant cells. Cells were maintained in medium with antibiotics.

### Stabilization of cell lines

Exponentially growing drug-resistant cells were harvested and resuspended in PBS supplemented with 3% FBS. N-Flag-δ or N-Flag-α4 subunit expression at the cell surface was detected using an anti-Flag M2 (Mouse anti-DYKDDDDK IgG (Clone M2), which binds the Flag epitope in any position) conjugated to SureLight^®^ APC, Columbia Biosciences, Frederic, MD). Cells were sorted at MGH Flow Cytometry Core facility using a BD 5 laser SORP FACS Vantage SE Diva system (BD Biosciences, San Jose, CA) using argon-ion laser excitation (633 nm, 35 mW). Emission for APC was detected at 650 nm. Dead cells and debris were excluded from the analysis by gating for intact cells using the forward and sideways scatter (488 nm, 320 mW). Uninduced cells were included as a negative control for autofluorescence. Data acquisition was carried out by analyzing 10 000 events/sample using CellQuest Software (BD Biosciences). FACS data were analyzed using FlowJo Software (Tree Star).

Following resorting of the cells in the pool in S1B Fig, one cell per well was seeded in a 96-well plate and cultured in antibiotic-enriched media. Some two dozen of these clones were analyzed for expression using flow cytometry and then by [^3^H]muscimol binding. The flow cytometry profile of the clone finally selected is shown in S1C & S1D Fig.

### Radioactive ligand binding

Radioactive ligand binding assays were carried out as previously described [13], with the exception that GABA_A_R binding sites were assayed at 250 nM [^3^H]muscimol because of this receptor’s higher affinity.

### Preparation of membrane, solubilization, immunoaffinity purification and reconstitution into CHAPS/asolectin micelles of receptors

Cells with inducible expression of N-Flag-α4β3 and α4β3N-Flag-δ were induced with tetracycline (1 μg/ml final concentration, Life Technologies) and 5 mM sodium butyrate (Sigma-Aldrich, St. Louis, MO) for 24 hours and harvested in 10 ml per ten 15 cm–dishes of 10 mM HEPES, 1 mM EDTA (buffer A) freshly supplemented with protease inhibitors and 1 mM PMSF (final concentration) at 4°C with all subsequent purification procedures carried out at 4°C. Cells were homogenized with three cycles of 10 s each separated by 30 s intervals (Ultra Turrax T25, IKA Works, Wilmington, NC), followed by 20 strokes in Kontes-Duval glass tissue grinder. Homogenate was centrifuged at 43,000 x g for 30 min at 4°C and the pellet was resuspended in the buffer A and the entire procedure repeated. The resulting pellet was resuspended in the buffer A and a final homogenization carried out by aspiration with a 21-gauge needle and expulsed through 27-gauge needle. Concentration of total protein was assessed with the Pierce BCA protein assay kit (Pierce, Rockford, IL). Under constant stirring, the membrane fraction was solubilized over 30 min by dropwise addition of 30 mM DDM (Anatrace, Maumee, OH) in buffer B (50 mM Tris/HCl, 150 mM NaCl, 2 mM CaCl_2_, 5 mM KCl, 5 mM MgCl_2_, 4 mM EDTA, pH 7.5) and 10% glycerol, supplemented with protease inhibitors and 1 mM PMSF, with the volume adjusted to produce an effective protein concentration of 1 mg/ml. Following an additional 2 hours of solubilization, the solution was centrifuged at 45,000 x g for 30 min at 4°C. Insoluble material in the pellet was discarded and the supernatant was stored in liquid nitrogen overnight.

After washing once with buffer B, 2 ml of Flag beads (Sigma-Aldrich) were pre-treated overnight at 4°C on polypropylene columns (Qiagen, Hilden, Germany) with 1.5 ml of 10 mg/ml poly-d-Lysine hydrobromide with gentle agitation. On the following day, beads were washed three times with six 2–ml volumes of buffer B with 10% glycerol and incubated with solubilized membrane fractions with gentle agitation for 2 hours. Once transferred back into propylene columns, beads were washed once with wash buffer (17 mM CHAPS, 8.5 mM asolectin in buffer B with 10% glycerol, homogenized with glass grinder and sonicated for 10 min), equilibrated with the same buffer for 1 hour with agitation and washed again. Two more washes with elution buffer (5 mM CHAPS, 200 μM asolectin in buffer B with 10% glycerol, prepared as with wash buffer) were performed prior to elution of receptors by incubation with two x 2–ml of elution buffer supplemented with 0.15 mg/ml of Flag peptide (Sigma-Aldrich) for 2 hours with gentle rocking (elution 1). Second elution (elution 2) and another overnight incubation with 2–ml (elution 3) followed. Preparations with 200 μM defined lipids (DOPC:DOPS:Cholesterol) instead of 200 μM asolectin produced comparable results.

Different protease inhibitor cocktails and their concentrations were assessed with regards to α4-subunit fragmentation: 1. 1:100 dilution of Protease inhibitor cocktail (Sigma-Aldrich); 2. addition of 60 nM DAPT, γ-secretase inhibitor (Tocris, Minneapolis, MN); 3. blend of 10 μg/ml Pepstatin A, 10 μg/ml Leupeptin, 10 μg/ml Chymostatin and 2 μg/ml Aprotinin (all from Sigma-Aldrich); 4. Complete protease inhibitor cocktail tablets at suggested or doubled concentration (Roche, Indianapolis, IN); 5. supplementation with 1 mM PMSF at membrane preparation stage or continuously throughout the procedure to address the short half-life of the compound. No reproducible and substantial differences were observed with regards to protease inhibitors used between individual preparations.

### Western blotting and deglycosylation

Purified protein was resolved on hand-casted 10% SDS-PAGE gels, loading approximately 10 μg of protein (as determined by Pierce BCA assay, Thermo Scientific, Rockford, IL) with 4x Laemmli buffer (Bio-Rad, Hercules, CA) supplemented with 10% β-mercaptoethanol (Sigma). Following transfer onto PVDF membranes (Immobilon-FL, Millipore, Billerica, MA) in 20% MeOH transfer buffer, membranes were washed with MeOH, air dried, re-wetted and blocked with Odyssey blocking buffer (PBS, Li-Cor, Lincoln, NE) for 1 hour at room temperature. Membranes were incubated overnight with the antibodies indicated in the figure legends, diluted in blocking buffer supplemented with 0.2% Tween-20, at 4°C with gentle rocking. Following washes with 0.1% Tween-PBS, membranes were incubated with secondary antibodies in blocking buffer supplemented with 0.2% Tween-20 and 0.01% SDS, for 1 hour at room temperature. Blots were imaged on an Odyssey Classic Infrared Imager (Li-Cor), images were quantified using the Rectangle tool in Image Studio Lite v5.2.5 (Li-Cor), by encompassing signal from both channels in a single oblong shape. Images were converted to gray scale for each channel separately and contrast and brightness adjusted uniformly for readout convenience. All antibodies used, along with their dilutions, are listed in S2 Table.

Deglycosylation of receptor subunits was carried out using PNGase F (NEB, Ipswich, MA) according to the manufacturer’s guidelines, and protein was resolved using 10% NuPAGE Bis-Tris gel (Life Technologies, Carlsbad, CA) in NuPAGE running buffer (Life Technologies), according to the manufacturer’s guidelines, with the remaining process as described before.

Coomassie Blue staining of the gels was performed on NuPAGE gels. Gels were fixed in 45% methanol, 10% acetic acid, 45% water (v/v/v), stained for 45 min in the same buffer supplemented with 0.125% Brilliant Blue R (Sigma) (w/v) and destained for 2–3 h using 40% methanol, 10% acetic acid and 50% water. Gels were dried and scanned.

### Electrophysiology

HEK 293 cells stably expressing tetracycline inducible α4β3N-Flag-δ or N-Flag-α4β3 GABA_A_ receptors were cultured on glass coverslips and induced with tetracycline (2μg/ml) 24 hours before recordings. The coverslips were placed in a recording chamber and perfused with the bath solution. Cells were voltage clamped at -50 mV using the patch clamp amplifier (Axopatch 200A, Molecular Devices, Sunnyvale, CA). GABA_A_ receptor-mediated chloride currents from HEK cells were recorded using the whole-cell configuration of patch-clamp electrophysiology. Data was acquired using Clampex version 8.1 (Molecular Devices), digitized at 10 kHz and filtered at 5 kHz. Current traces were analyzed using Clampfit version 9 (Molecular Devices). Series resistance ranged from 0.5–4 MΩ and cell capacitances from 9–18 pF. Membrane-capacitance and series-resistance were compensated electronically by > 85% with a lag of 10 μ seconds. Drugs were delivered via a multichannel superfusion pipette coupled to a piezoelectric elements that switched superfusion solution in < 1 millisecond [17]. The recording chamber was continuously perfused with the bath solution in mM: 145 NaCl, 5 KCl, 10 HEPES, 2 CaCl_2_, and 1 MgCl_2_, 10 glucose, pH 7.4 (pH adjusted with N-methyl glucosamine). The pipette solution for whole-cell recordings contained in mM: 140 KCl, 10 HEPES, 1 EGTA and 2 MgCl_2_ at pH 7.3 (pH adjusted with KOH). Open pipette resistances ranged from 1.6–3 M. Errors are standard deviation from at least three replicas.

### Statistical analysis

Statistical analysis for electrophysiology was done using GraphPad Prism 6. The GABA EC_50_ was determined using Origin 6 software (OriginLab). Analysis of the [^3^H]muscimol binding was carried out using Igor Pro 7 (WaveMetrics). For Western blotting, the ratio of the signal intensity of individual antibodies for each sample was determined. Pooled (weighted) mean and standard deviation was determined for each receptor and p value determined by two-tailed unpaired Student’s t-test, using GraphPad QuickCalcs software.

## Results and discussion

### Development of a stable, inducible HEK293-TetR α4β3N-Flag-δ GABA_A_R cell line

The ratio of transfected cDNAs encoding individual GABA_A_R subunits can affect stoichiometry of δ–subunit–containing pentamers, therefore affecting the EC_50_ of the GABA concentration-response curve. In some cases, Hill coefficients of less than one have been reported, indicating heterogeneity [8, 10, 18].

We chose to transfect HEK293-TetR cells with constructs in the weight ratio of α4:β3:δ 2:1:0.25, which was shown to produce stoichiometry of (α4)_2_(β3)_2_(δ)_1_ in a systematic study assessing this relationship by Western blotting of α-bungarotoxin tagged subunits [11]. Our reasoning was further supported by the reported elevated stability of the δ-subunit [10]. We placed a Flag-tag on the δ–subunit so that affinity purification would yield only those expressed heteropentamers that contained δ–subunits. The cell line expressing α4β3, with Flag-tag on the N-terminal of α4-subunit, was constructed in analogous manner to serve as a control in the experiments.

HEK293 Tet-R cells transfected with aforementioned ratio of subunits underwent antibiotic selection and FACS sorting using a fluorophore-conjugated antibody against the Flag-tagged δ-subunit. Individual cells were collected to establish monoclonal populations in a 96-well format (S1 Fig). Flow cytometry analysis allowed us to identify several candidate clones with high expression of the protein of interest, which were subsequently screened in a functional [^3^H]muscimol binding assay. The clone used in this study yielded 19.1 ± 3.1 pmol of [^3^H]muscimol binding sites per mg of membrane protein (n = 17). This is comparable to that of the inducible α1β3γ2L cell line we previously reported [13].

The specific activity of receptors in cell membranes increased over 48 hours of induction time, reaching ~30 pmol/mg of total membrane protein. However, we routinely induced for 24–30 hours in order not to stress the cells. For the five inductions averaged in Table 1, the yield in the harvested cell membranes was 75 ± 9 pmol/plate with a specific activity of 17.2 ± 2.8 pmol [^3^H]muscimol binding sites/mg of membrane protein.

**Table 1:** Representative yield of α4β3N-Flag-δ GABA_A_R purification by anti-Flag affinity chromatography.

Purification fractions	Total specific [^3^H]muscimol binding sites (pmol) ^a^	% overall yield ± standard deviation ^b^	Average [3H]muscimol specific activity ^b^
Starting membranes	4577	100	17 ± 2.8 pmol / mg
Solubilized supernatant Applied on column	3927	80 ± 4.3	12 ± 1.5 pmol / ml
Flow through during binding	837	23 ± 3.0	3.6 ± 0.82 pmol / ml
First wash flow through	67	1.2 ± 0.21	2.1 ± 1.1 pmol / ml
Elution 1 (E1)	723	13 ± 1.4	54 ± 12 pmol / ml
E2	188	3.0 ± 1.3	24 ± 13 pmol / ml
E3	203	3.1 ± 1.0	26 ± 10 pmol / ml
Total elution	1114	20 ± 3	39 ± 12 pmol / ml

^a^Total amount of GABA_A_R, measured as specifically bound [^3^H]muscimol (250–500 nM) sites from one representative purification using 60 plates of cells.

^b^ Mean ± standard deviation of five purifications.

### Electrophysiological and pharmacological characterization of the α4β3N–Flag–δ GABA_A_R cell line

We first characterized this GABA_A_R expressed in the plasma membrane 24–30 hours after induction. Because α4β3δ receptors expressed in oocytes often appear to have different gating kinetics such as low Hill coefficient and very low EC_50s_ [7, 8], we will only compare the properties of our cell line’s receptors, wherever possible to those in other mammalian expression systems.

**Fig 1:**
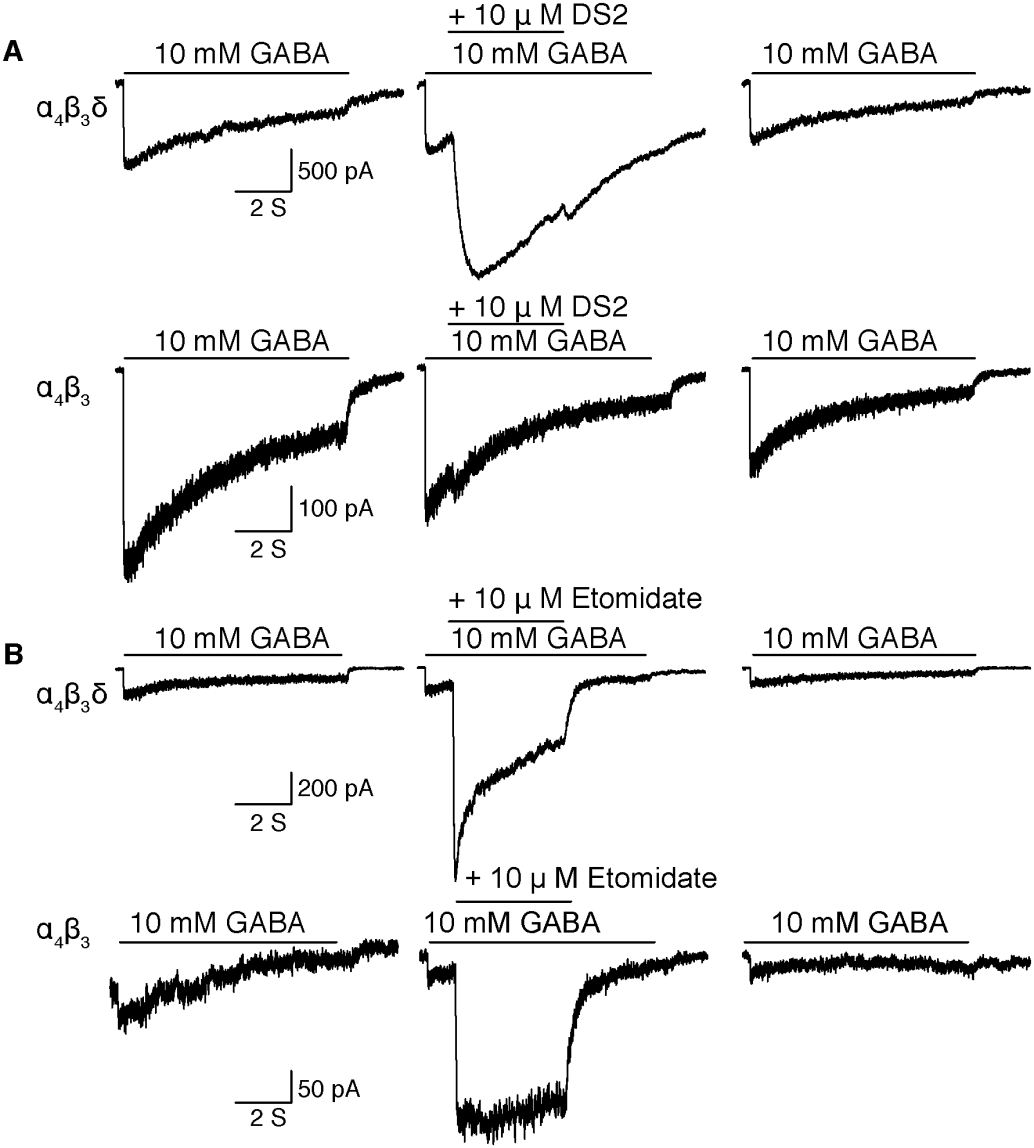
The δ-subunit is expressed in the α4β3N–Flag–δ GABA_A_R stable cell line. Representative current traces show the effect of DS2 (**A**) and Etomidate (**B**) on 10 mM GABA–elicited currents on α4β3N–Flag–δ (upper panel) compared to N–Flag–α4β3 (lower panel). Currents were elicited in a notch protocol by an eight second pulse of GABA, during which drug was co-applied for 4 seconds 1 second after the GABA perfusion started. Concentrations are indicated in the figure.

DS2 is a specific positive allosteric modulator of δ-subunit containing receptors [6], therefore, to confirm the incorporation of δ-subunits in the GABA_A_R complex, we tested DS2 modulation of GABA_A_R mediated currents (Fig 1A). We employed a notch protocol herein, characterized by short co-application of drug one second after GABA application (Fig 1A; middle column). In cells expressing α4β3N–Flag–δ receptors, DS2 (10 μM) enhanced maximum GABA elicited currents by 230 ± 63% (SD, n = 6; Fig 1A, top panel); whereas, in cells expressing only N–Flag–α4β3 receptors, DS2 produced only a mild enhancement of 12 ± 7% (n = 6; differs from α4β3N–Flag–δ with p = 0.0001; Fig 1A, bottom panel). Therefore, these data confirmed the incorporation of the δ-subunit in the GABA_A_R complex in the α4β3N–Flag–δ GABA_A_R cell line.

It is a characteristic of α4β3δ GABA_A_Rs that GABA acts as a partial agonist, opening only a fraction of the available channels (i.e. it has a low P_open_) [19, 20]. Using the above notch protocol, etomidate (10 μM) enhanced the maximum GABA elicited currents by 540 ± 235% (n = 7; Fig 1B, top panels) in cells expressing α4β3δ receptors. This experiment provides an estimate of the efficacy of GABA. Thus, if we assume that co-application of 10 mM GABA with 10 μM etomidate opens 100% of available channels, then 10 mM GABA opened only 17 ± 6% (n = 7) of available channels, indicating GABA is a low efficacy agonist of the α4β3N–Flag–δ receptors, as has been previously reported for α4β3δ GABA_A_Rs [9, 20, 21]. In the control N–Flag–α4β3 cell line, the etomidate enhancement was similar (490 ± 84%, n = 4; Fig 1B, lower panel) suggesting that inclusion of the δ-subunit does not change GABA’s efficacy. On the other hand, introduction of the δ-subunit into α4β3 receptors did dramatically increase the rate of current decay during the 4-s co-activation of GABA with etomidate (Fig 1B, middle panels).

**Fig 2:**
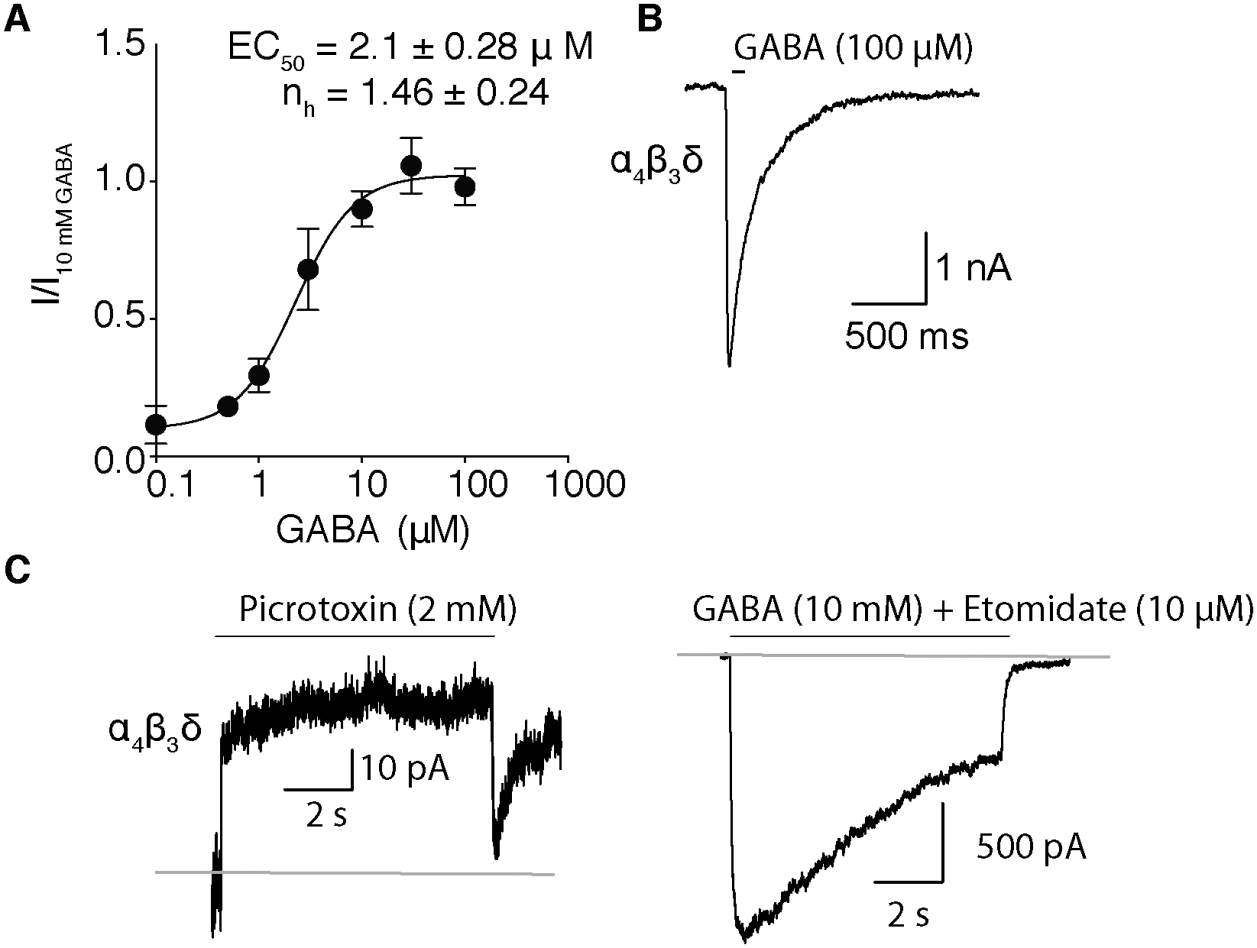
The gating properties of α4β3N–Flag–δ GABA_A_Rs. (**A**) GABA concentration-response curve for α4β3N–Flag–δ receptor mediated currents. Currents were elicited by application of varying concentrations of GABA (0.1–10 mM). Peak current amplitudes in each cell were normalized to that obtained with 10 mM GABA. (**B**) Representative current trace obtained by application of an 8.5 ms pulse of 100 μM GABA to measure the deactivation rate. (**C**) α4β3N–Flag–δ receptors are spontaneously open. (Left panel) Representative trace of outward currents observed by application of 2 mM Picrotoxin to inhibit the spontaneously open receptors. (Right panel) The estimate of the maximum inward currents obtained by co-application of 10 mM GABA with 10 μM Etomidate to gate all available receptors. The gray lines are drawn by eye to represent the baseline. At least three cells per concentration were used throughout experiments.

The half-stimulatory GABA concentration (GABA EC_50_) was determined by normalizing peak current amplitudes obtained by the application of eight-second pulses of varying concentrations of GABA (0.1–100 μM) to the peak amplitude value obtained with 10 mM GABA for the same cell (S2 Fig). The data was plotted against the GABA concentration and fitted with the Hill equation to yield a GABA EC_50_ value of 2.1 ± 0.28 μM and a Hill coefficient of 1.5 ± 0.24 (n = 3–5 cells at each point; Fig 2A), similar to previous values reported for δ–containing receptors in mammalian cells [9, 22, 23].

The kinetics of the activation phase of currents were well fit by a single exponential whose time constant, τ, decreased with increasing GABA concentration, from 165 ± 20 ms at 0.1 μM GABA (n = 3) to 6 ± 1 ms at 100 μM GABA (n = 3), above which (0.3–10 mM) there was no increase, probably because of our limited solution exchange rate of 2–5 ms. The initial rate was linear up to ~10 μM and could be fit to the rate equation

$$k_{Act}=k_{-1}+\left(k_{+1}\times \left[GABA\right]\right),$$

where k_Act_ is the measured activation rate following addition of GABA (1/τ), and k_+1_ and k_–1_ are the apparent forward and reverse GABA binding rates. A plot of these data yielded k_+1_ = 5.7 ± 0.2 x 10^6^ s^–1^M^–1^ and k_–1_ = 5 ± 1 s^–1^, yielding an apparent dissociation constant of 0.9 ± 0.2 μM. In α4β3γ2 receptors on the other hand, k_+1_ was 6 x 10^6^ s^–1^M^–1^, a value similar to our value for α4β3δ receptors, but k_–1_ was 20-fold faster (100 s^–1^) than in α4β2δ receptors [21]. This accounts for the 20-fold higher apparent dissociation constant and GABA EC_50_ characteristic of GABA_A_Rs with a γ-subunit.

In a recent study of α1β3δ GABA_A_Rs in HEK cell lines, Botzolakis, *et al*. [10] reported 10–90% rise times at 1 mM GABA ranging from 4–22 ms depending on the construct ratio. We note however that all these rise times are considerably faster than ~100 ms at 100 μM GABA previously reported by Brown et al in α4β3δ receptors [22]. Although their solution exchange times were comparatively slow, 20–30 ms, the discrepancy might also be caused by the way the cell lines were constructed, which leaves uncertainties about the stoichiometry [22].

The rate of deactivation (channel closing) was measured after an 8.5 ms pulse of 100 μM GABA, a time chosen to maximize current and minimize the contribution of desensitization before GABA is withdrawn (Fig 2B). At 100 μM GABA, the closing phase of the current was best fit with two exponentials that had time constants of 1100 ± 440 and 175 ± 50 ms (6 cells). The fastest phase occupied 76 ± 12% of the total current decay amplitude. The deactivation kinetics were independent of GABA concentration because similar values were obtained at 10 mM GABA. Our values may be compared to that of ~400 ms reported by Brown et al in α4β3δ receptors using a lower time resolution perfusion system [22]. In another study of α1β3δ receptors, the deactivation rate varied somewhat with the ratio of constructs used in the transfection from 100–200 ms; at a ratio of 1:1:0.3, the closest condition to ours, their value was ~170 ms, not different from our faster value [10]. Using the concatenated subunits β3-α1-δ/β3-α1, Liu et al. found a deactivation time constant of ~50 ms [24].

The high density of GABA_A_Rs that can be achieved in our inducible cell line yields larger currents that allow spontaneous activity to be clearly observed. Thus, application of 2 mM picrotoxin, a specific blocker of GABA_A_ receptors, for 8 seconds revealed an outward current, suggesting that spontaneously open receptors were being inhibited (Fig 2C). The fraction of spontaneously open receptors was estimated to be about 3.2 ± 0.6%, by assuming that the outward current activated by an 8-s pulse of GABA (10 mM) co-applied with etomidate (10 μM) opened all the receptors expressed in the same cells after washout of PTX (Fig 2C, right panel). This small amount of spontaneous activity is consistent with a single channel study in which HEK cells were transiently transfected with rat or human α4:β3:δ cDNAs in the ratio 1:1:1 [25].

The desensitizing phase of the currents during an 8-sec exposure to GABA (1–100 μM) was best fit by a single exponential and did not vary significantly with GABA concentration. A desensitization time constant, τ, of 2.6 ± 0.9 s was obtained when times from all GABA concentrations were averaged. Brown et al reported a τ of 4.8 s at 100 μM GABA in their α4β3δ cell line [22]. The degree of desensitization was different from that reported by Botzolakis et. al. for α1β3δ receptors [10]. Whereas in our δ cell line we observed ~70% desensitization in 4 seconds at 100 μM GABA, the latter workers observed little desensitization in 4 seconds at transfection ratios similar to ours, but 40–70% at much lower transfection ratios. In the concatenated subunits β3-α1-δ/β3-α1, desensitization after 4 s at 1 mM GABA was ~40% with a time constant of 4.7 s [24].

### Solubilization, purification and reconstitution of the α4β3δ GABA_A_Rs into CHAPS/lipid micelles

Receptors were purified following the procedure of Dostalova et al. with minor modifications discussed below [12, 13]. An SDS-PAGE gel loaded with samples from each stage of the procedure illustrates the progress of the purification (S3 Fig). In one purification (Table 1, column 2), membrane fractions extracted from cells grown on sixty 15–cm plates containing 4.6 nmol of [^3^H]muscimol binding sites yielded 1.1 nmol of purified receptor. In the average of five purifications (Table 1, columns 3 & 4), 20% of binding sites were lost during membrane solubilization with 30 mM DDM and a further 23% was not retained on anti-Flag affinity chromatography beads. After washing, purified receptors were equilibrated in 5 mM CHAPS and 200 μM lipid. The nature of the lipid had no major influence on the purified receptor’s properties. The lipids used were asolectin, DOPC:DOPA:cholesterol or DOPC:DOPS:cholesterol in the molar ratio 52:15:33. Following three consecutive elutions with 0.15 mM Flag peptide, the combined average yield of receptor was 20%, corresponding to 0.9 ± 0.2 nmol per ml of [^3^H]muscimol binding sites from 60 plates with specific activity of 39 ± 12 pmol per ml of combined eluted fractions.

### Characterization of α4β3N-Flag-δ GABA_A_R by radioactive ligand binding assays

**Fig 3:**
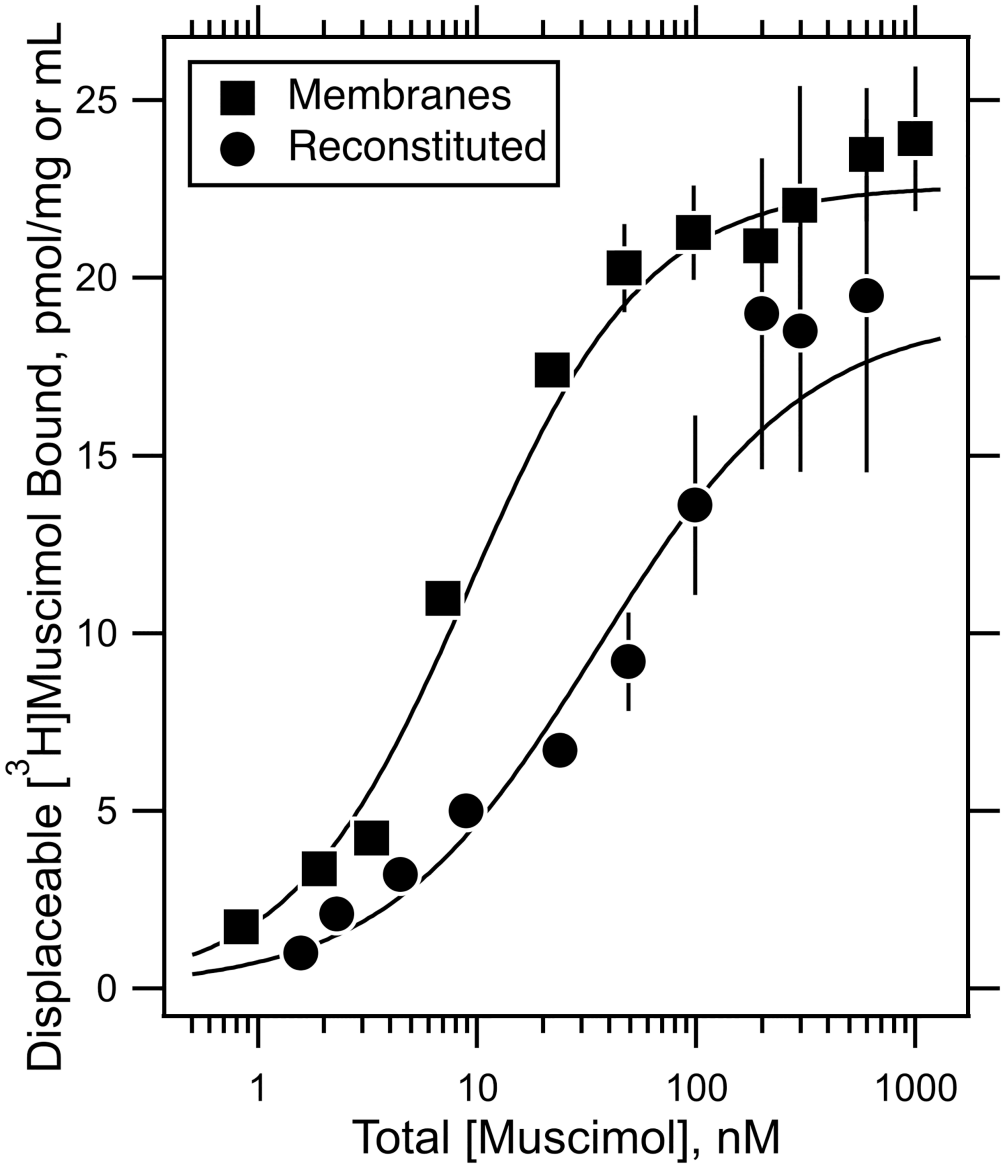
The binding isotherm of the agonist [^3^H]muscimol to the α4β3N-Flag-δ GABA_A_ receptor in native membranes and reconstituted into CHAPS/lipid micelles. Binding curves of [^3^H]muscimol to α4β3N–Flag–δ GABA_Α_Rs, both, in cell membranes (pmol/mg membrane protein) and after purification and reconstitution into micelles of 5 mM CHAPS and 200 μM DOPC:DOPS:Cholesterol in mole ratio 52:15:33 (pmol/mL). Displaceable binding was determined as the difference between binding in the presence and absence of 1 mM GABA using a filtration assay in triplicate. The displaceable binding and its standard deviation was determined by subtracting these two values and propagating errors at each total muscimol concentration. The curves were fitted by nonlinear least squares with weighting by standard deviation. These yielded apparent dissociation constants of 9.2 ± 0.6 and 35 ± 12 nM, respectively. The B_max_ of the membranes was 22.6 ± 0.5 pmol/mg and for reconstituted receptors in micelles was 19 ± 3 pmol/mL. The Hill coefficients differed little from one (1.07 ± 0.3 and 0.90 ± 0.05 respectively).

Binding of the GABA_A_R agonist [^3^H]muscimol to cell membranes was displaceable by GABA. The displaceable binding increased with [^3^H]muscimol concentration reaching a plateau at about 100 nM (Fig 3). The data were fitted by nonlinear least squares to the Hill equation, yielding an EC_50_ of 9.2 ± 0.6 nM, a Hill coefficient of 1.1 ± 0.03 and 22.6 ± 0.5 pmol of sites per mg of membrane protein. This apparent affinity is respectively equal to and 5–fold higher than in our previously developed α1β3 and α1β3γ2 GABA_A_R cell lines [13, 26]. A similar 5–fold differential has been noted previously in a study in which GABA_A_Rs containing either γ- or δ-subunits were selectively immuno-precipitated from rat brain, although the absolute values were smaller than ours [27].

After receptors were reconstituted into CHAPS/DOPC:DOPS:Cholesterol micelles they exhibited a higher apparent dissociation constant of 35 ± 12 nM compared to membranes (Fig 3). A preliminary value in CHAPS/asolectin also yielded a higher value compared to membranes [28]. Such a difference between membranes and micellar receptors has been noted before [13], and we suspect it comes from a modulatory action of CHAPS that favors a lower affinity conformation.

**Fig 4:**
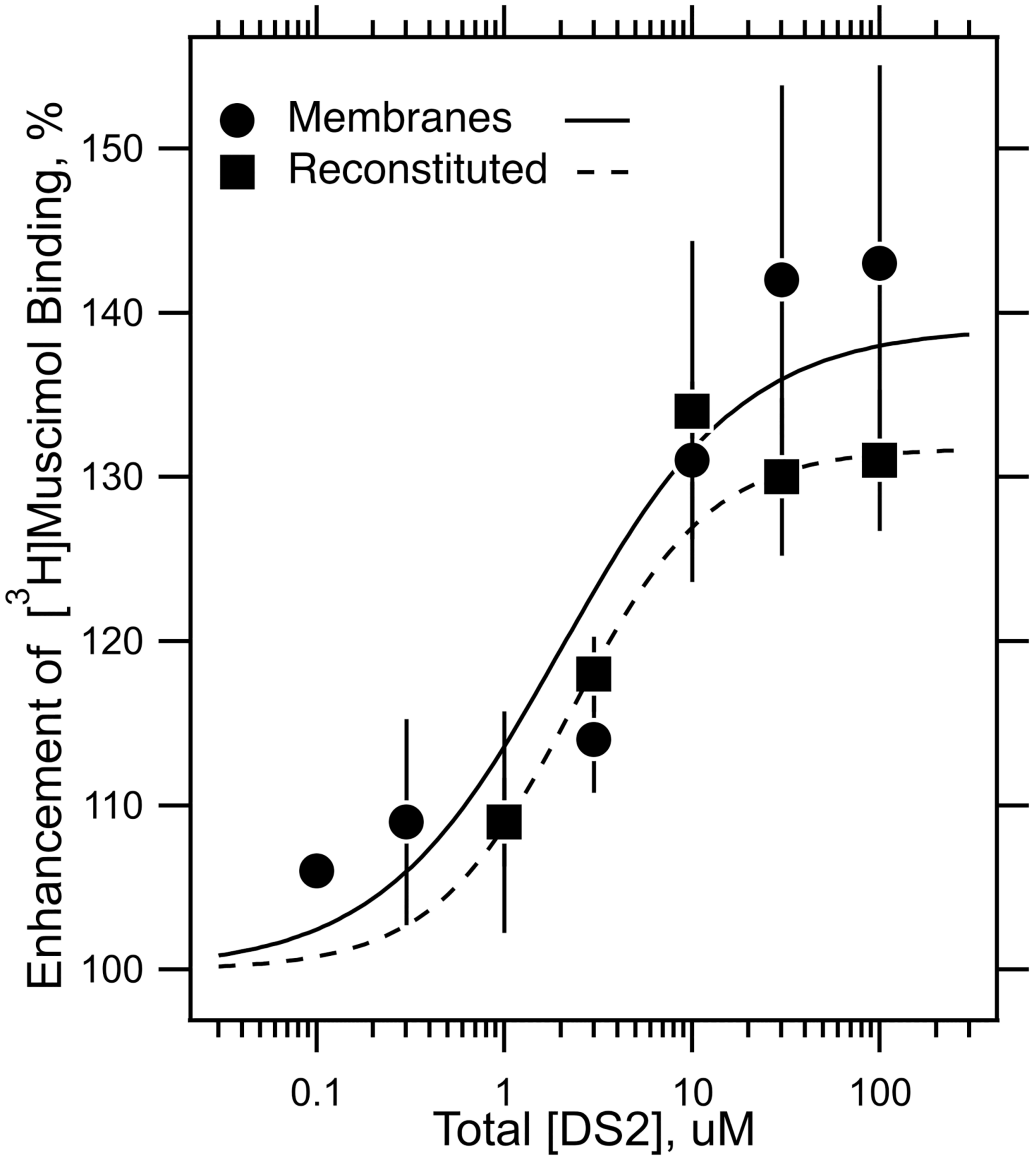
DS2 enhances agonist binding in δ-subunit containing receptors. The δ-subunit specific modulator, DS2, modulates [^3^H]muscimol binding (2 nM) in α4β3N-Flag-δ GABA_A_Rs. For the membranes, the data are the mean and standard deviation of two experiments, and for micelle reconstituted receptors for a single experiment in triplicate. The curves were fitted by nonlinear least squares to the Hill equation. The EC_50_ (μM), Hill coefficient and maximum modulation were: for membranes, 2.0 ± 0.7 μM, 0.9 ± 0.2, 139 ± 4%; for reconstituted 2.3 ± 0.8 μM, 1.2 ± 0.5, 132 ± 4%.

The δ-subunit specific agent DS2 at 30 μM enhanced displaceable [^3^H]muscimol (2 nM) binding by 142 ± 12% (n = 10) in membranes and 128 ± 11% (n = 7) in GABA_A_Rs reconstituted into CHAPS/lipid micelles (Fig 4). In a parallel reconstitution on the same day into CHAPS/asolectin or CHAPS/DOPC:DOPS:cholesterol, no difference in degree of enhancement was found. The concentration–dependence of DS2 modulation of 2 nM [^3^H]muscimol binding yielded an EC_50_s of 2.0 ± 0.7 μM for receptors in native membranes and 2.3 ± 0.8 μM for receptors reconstituted into CHAPS/PC:PS:Cholesterol micelles (Fig 4).

### Biochemical characterization of the CHAPS/asolectin reconstituted α4β3N-Flag-δ GABA_A_ receptor

#### All subunits are glycosylated

**Fig 5:**
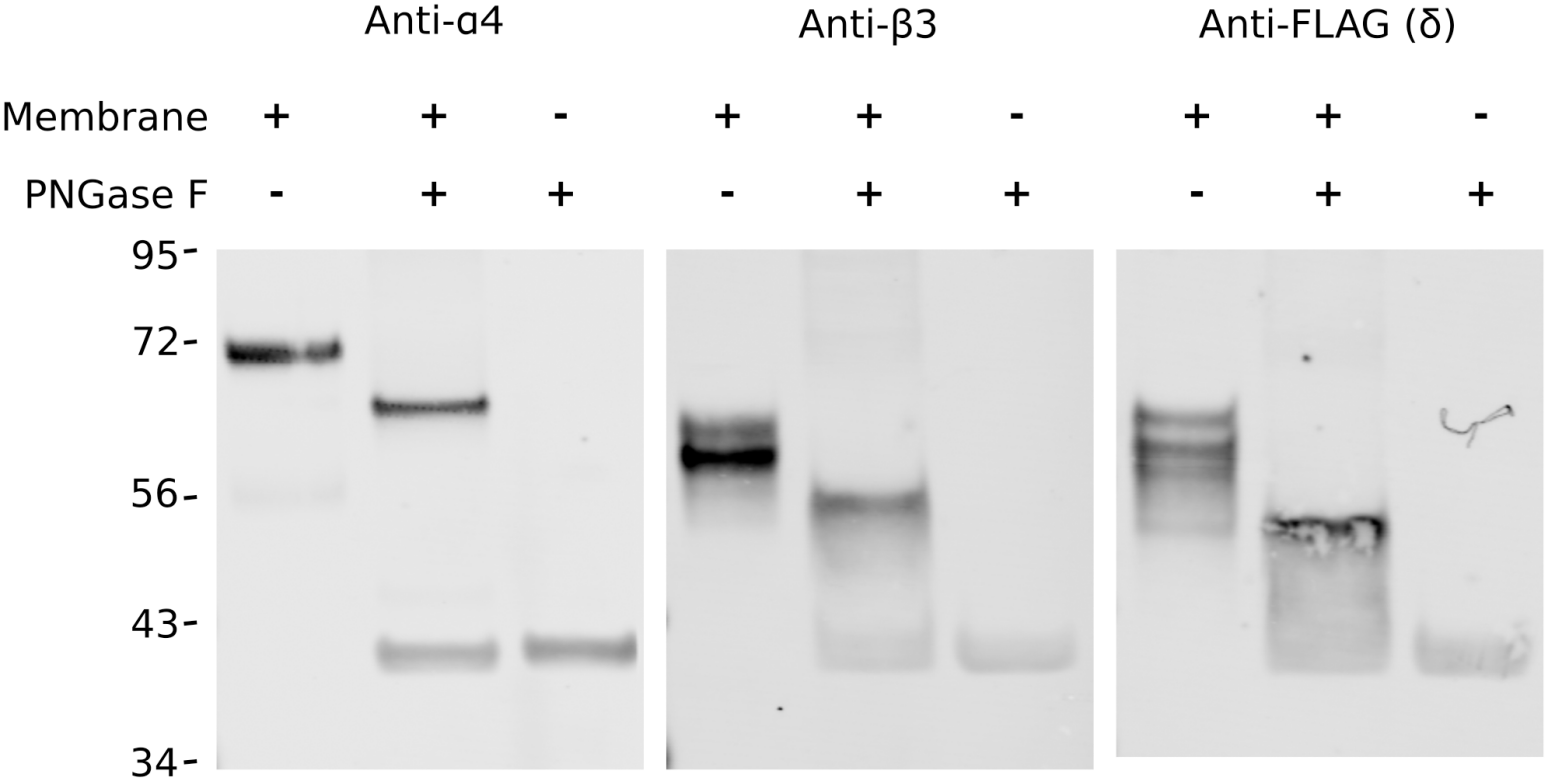
All subunits of the α4β3N-Flag-δ GABA_A_ receptor are glycosylated. Purified receptors were resolved by Western blotting with antibodies for α4- and β3-subunits or Flag, as shown on the left, middle and right panels, respectively. In each of the three panels, reading left to right the lanes are: purified receptor; purified receptor after deglycosylation with PNGase F, and PNGase F alone. The band below the 43 kDa marker was nonspecific and associated with PNGase F. Numbers on the left side indicate MW in kDa. The α4 and β3 antibodies are polyclonal. Brightness and contrast were uniformly adjusted for each panel.

Analysis of the purified and reconstituted α4β3N-Flag-δ receptor into CHAPS/asolectin micelles by the means of SDS-PAGE followed by Western blotting indicated the presence of all three subunits as expected, however the observed molecular weight of each subunit was larger than predicted by the mature amino acid sequence. The α4-subunit presented at 72 and 54kDa (expected at 66kDa), the β3-subunit presented as double band pattern of approximately 62 and 58kDa (predicted at 52kDa) and the δ-subunit appeared as a ladder of three bands around 58kDa (predicted at 49kDa). This shift in mobility can be associated with post-translational modification in the form of an N-linked glycosylation commonly described for the GABA receptors [29]. Indeed, after *in vitro* deglycosylation of the purified receptors by the relatively nonspecific PNGase F (Fig 5), the molecular weight of each subunit decreased by approximately 5–10 kDa.

#### Origin of α4 fragmentation

**Fig 6:**
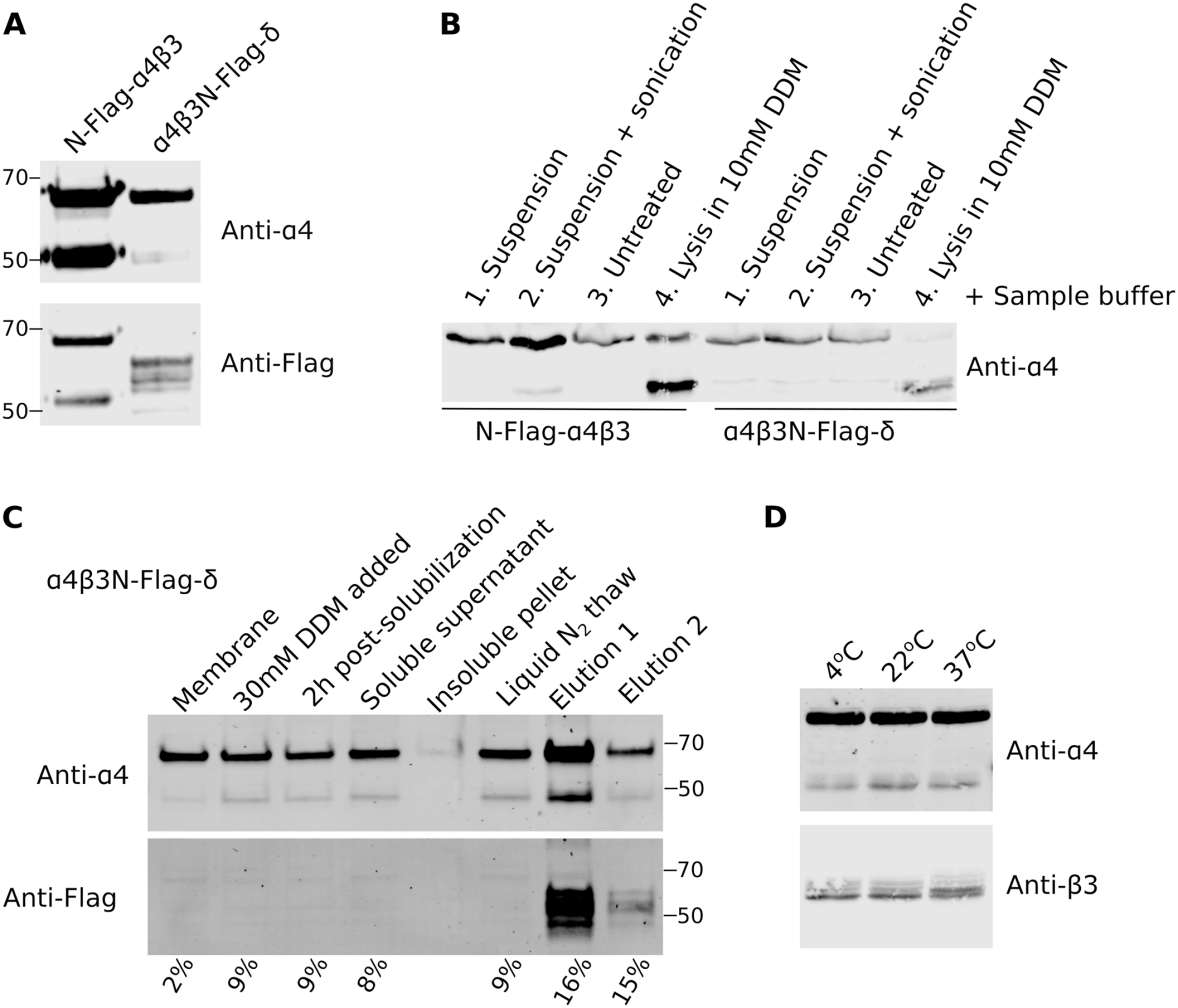
Stability of the α4-subunit. (**A**) Western blot depicting fragmentation of α4-subunit seen as two bands in N-Flag-α4β3 and α4β3N-Flag-δ receptors reconstituted into CHAPS/asolectin micelles is presented. Both α4 bands are identified by polyclonal anti-α4 and monoclonal anti-Flag antibodies in the former receptor, confirming identity of the band. Numbers on the side indicate the position of the molecular weight markers (kDa). (**B**) Cells induced to express indicated GABA_A_Rs were prepared for Western blotting by either 1. suspending cells in suspension buffer; 2. suspending and sonicating; 3. leaving in a monolayer (untreated); or 4. lysing directly in the well with suspension buffer supplemented with 10 mM DDM; as indicated, prior to lysing cells with a 4x Laemmli sample buffer with 10% β-mercaptoethanol. Suspension buffer was supplemented with Protease Inhibitor Cocktail (Sigma) at 1:100 dilution. (**C**) Representative Western blot of samples obtained during α4β3N-Flag-δ receptor purification, as described in the materials and methods section. Numbers under each lane indicate the fraction the lower band comprises of the higher band, expressed as percentile points. (**D**) Membrane fraction from the α4β3N-Flag-δ was incubated for 1 hour at indicated temperatures prior to analysis by Western blotting. All blots are presented as grayscale and were uniformly adjusted for brightness and contrast to facilitate analysis. Full immunoblots used to make panels A-D are presented as S4–S7 Figs.

The appearance of two bands upon immunoblotting for α4 has been reported previously [30]. We have reported previously that the identity of these bands was confirmed by the LC/MS/MS [28]. Furthermore, Western blotting of the CHAPS/asolectin reconstituted N-Flag-α4β3 receptor with anti-Flag and anti-α4 antibodies produced matching bands (Fig 6A and S4 Fig). The extent of α4 protein fragmentation, measured as a ratio of lower to higher molecular weight bands, varied greatly between independent protein preparations from 2% to 125% (n = 6). When cells were lysed with 4xLaemmli buffer upon suspension or directly in the well, no fragmented protein was observed (lanes 1 and 3), unless plasma membrane was previously disrupted by sonication or 10 mM DDM treatment (lanes 2 and 4; Fig 6B and S5 Fig). Fragmentation could be observed already during the preparation of the plasma membrane fraction and sometimes occurred during the micellar reconstitution process (Fig 6C and S6 Fig). This observation implies that the α4-subunit is synthesized intact and that fragmentation occurs during processing. However, we observed no correlation between protein fragmentation and the concentration or spectrum of protease inhibitors used throughout the purification process. Furthermore, incubation of the plasma membrane fraction at temperatures ranging from 4 to 37°C for 1 hour did not have any effect on protein fragmentation, suggesting that it is not an effect of enzymatic activity (Fig 6D and S7 Fig). Our previous work on α1β3 and α1β3ɣ2 receptors utilized the same purification procedure without any fragmentation of the α1-subunit, suggesting that the large cytoplasmic loop of the α4-subunit is the problem (70 residues in α1 subunit vs. 161 in α4). While this is unfortunate, it is quite possible that the pentamer remains intact. For example, crystallization of the homopentameric serotonin 5-HT_3A_ receptor was aided by cleavage in the extracellular domain, yet the C-terminal transmembrane domain helix M4 remained part of the structure [31].

### Estimating the stoichiometry of α4β3N-Flag-δ GABA_A_Rs

The exact subunit stoichiometry of GABA_A_R pentamers containing the δ-subunit remains uncertain, and indeed during development of this cell line it became more likely that several stoichiometries may be possible [10]. Although our receptor was not optimally designed for stoichiometry determinations, we attempted to place some limits by utilizing the duplex capabilities of the near-infrared fluorescence detection system and comparing the ratio of two antibodies both in the N-Flag–α4β3 receptor and in the α4β3N-Flag–δ receptor after purification. As polyclonal anti-α4 and monoclonal anti-Flag antibodies recognize the same subunit in the former receptor, the observed signal ratio can be normalized to 1, allowing the molar ratio of α4 to δ to be determined in the latter receptor (S8 Fig). The α4: Flag(δ) ratio for N-Flag-δ containing receptors was below 1 (0.40 ± 0.28 SD, n = 5) compared to 1 (± 0.11 SD, n = 5) in N-Flag-α4β3, with p = 0.0073 (two-tailed unpaired Student’s t-test). However, because of the amount of processing required before quantifying the blots, there are likely to be systematic errors that are not included in the above statistics. Nonetheless, it is safe to conclude that there are not two α-subunits for each δ-subunit. This is consistent with the evidence that etomidate photolabels a site in the transmembrane domain between two β subunits in receptors from this same cell line [28].

Further characterization was less secure, requiring the comparison of the α4β3N-Flag–δ and the N-Flag–α4β3 receptors using a β3 monoclonal and an α4 polyclonal antibody. We found that the ratio of β3: α4 in α4β3N-Flag- δ was 1.68 (± 0.15 SD, n = 4) times higher than in N-Flag-α4β3 (S9 Fig). If we assume a stoichiometry (α4)_2_(β3)_3_ [11], then the measured ratio of β3: α4 in the α4β3N-Flag–δ receptor is 2.5 (1.68 x 3/2), which is not inconsistent with the results in the preceding paragraph and suggests a possible stoichiomtery of (α4)_1_(β3)_3_(δ)_1_. Other assumptions lead to different answers, so this assignment is not definitive. Nor can we rule out that there might be more than one pentamer stoichiometry present, although our functional titrations offer no hint (Figs 2 & 3). Indeed, the concept of variable stoichiometries has been suggested by several groups [10, 11, 32, 33], and was clearly observed by Wagoner and Czajkowski [11]. We conclude that structural studies will be required to resolve the stoichiometry.

## Conclusion

We have established an inducible cell line that yields α4β3δ GABA_A_ receptors at high levels suitable for biophysical and biochemical studies. Our characterization shows the receptors in this cell line to have the gating and pharmacological properties to be expected of a GABA_A_R of this subunit composition. We performed a relatively detailed analysis of gating in order that these properties may be linked to structural studies that this cell line may eventually promote.

We have demonstrated that this receptor can be purified in CHAPS/lipid micelles while maintaining equilibrium allosteric interactions between the agonist and anesthetic binding sites in large enough quantities to enable studies of structure, dynamics and function. Such studies may definitively resolve many of the uncertainties that cannot be resolved by electrophysiological techniques alone. The strategy of using the Tet-R inducible cell line, which was introduced by the Khorana laboratory to study rhodopsin [16], to study large heteropentameric ligand-gated ion channels [12, 13] has already yielded a crystal structure of the 5HT_3_ receptor and a dynamics study of a GABA_A_R in the vicinity of the etomidate–binding site [31, 34].

## Supporting information

S1 Fig Stages of establishing monoclonal HEK293 TET-R α4β3N-Flag-δ stable cell line. Panels A-D depict flow cytometry results at indicated stages of cell line development. (**A**) Transfected cells following antibiotic selection. (**B**) Cells as in panel A following 24 hours of tetracycline induction. (**C**) Monoclonal population of cells with the highest expression of N-Flag-δ used in the study, confirmed to have approximately 19 pmol/mg [3H]muscimol binding sites in the membrane fraction. (**D**) Histogram of cells in panel C.
(TIF)

S2 Fig Representative normalized currents elicited by varying concentrations of GABA on α4β3N-Flag-δ expressing cells. Currents were recorded using whole cell patch clamp technique from cells exposed to 0.1; 0.5; 1; 3; 10; 30; 100 μM of GABA for 8 seconds. Each concentration was recorded on 3–5 cells and peak amplitude was normalized to the recording obtained with 10 mM GABA on the same cell. Representative current for a single cell is shown.
(TIF)

S3 Fig Coomassie Blue staining of the SDS-PAGE. Samples collected throughout the purification procedure were resolved on 10% SDS-PAGE gel under denaturing conditions. Second lane (membrane) was loaded with 30 μg (0.86 pmol) of total membrane protein. Gel was subsequently fixed and stained with Coomassie Blue. A similar pattern was observed by Chiara and colleagues in photolabelling studies (Chiara *et al*., *2016)*. The main bands observed in the elution fractions represent individual subunits of the α4β3δ GABA_A_ receptor. Numbers to the right represent molecular weights of the markers. Number of pmols in the Elution fractions loaded was determined by [^3^H]muscimol binding assay. Contrast and brightness of the bottom panel were adjusted uniformly to facilitate inspection.
(TIF)

S4 Fig The α4-subunit is fragmented. Whole immunoblot used to produce Fig 6A is presented. Two independent preparations for each reconstituted receptor is presented and those additional preparations flank the lanes shown in Fig 6A. Molecular weight standards are shown on the left hand side including their size in kDa. Lower two panels are greyscale representation of individual channels corresponding to immunoblot with antibodies as indicated.
(TIF)

S5 Fig The protein preparation procedure affect α4-subunit fragmentation. Whole immunoblot used to produce Fig 6B is presented. Immunoblot with polyclonal anti-α4-subunit antibody produced additional band above 70 kDa. This band was not observed on other occasions and it did not correlate with anti-Flag immunoblot on samples positive for N-Flag-α4β3, suggesting it to be non-specific.
(TIF)

S6 Fig Fragmentation of α4-subunit throughout the standard purification procedure. Whole immunoblot of α4β3N-Flag-δ receptor used to produce Fig 6C is presented. Molecular weight markers are flanking with their respective size in kDa indicated on the left hand side.
(TIF)

S7 Fig Incubation of protein lysate at different temperatures does not affect α4-subunit fragmentation. Whole immunoblot used to produce Fig 6D is presented. The molecular weight marker is shown on the left, with respective size indicated on the right hand side. The marker migrated at a slight angle.
(TIF)

S8 Fig Representative anti-α4 and anti-Flag duplex Western blot. Reconstituted N-Flag-α4β3 and α4β3N-Flag-δ receptors from two independent purifications of each were analyzed by Western blotting. The bottom (color) panel represents the original membrane scan that was used for quantification. Grayscale panels are depicted to facilitate analysis. Immunoblots were uniformly adjusted for brightness and contrast.
(TIF)

S9 Fig Representative anti-α4 and anti-β3 duplex Western blot. Elution 1 and elution 2 fractions of the reconstituted N-Flag-α4β3 and α4β3N-Flag-δ receptors were analyzed by Western blotting. The bottom (color) panel represents the original membrane scan that was used for quantification. Grayscale panels are depicted to facilitate analysis. Immunoblots were uniformly adjusted for brightness and contrast.
(TIF)

S1 Table Primers used in the study. (DOCX)

S2 Table Antibodies used in the study. (DOCX)

S1 Text Subunit amino acid sequences used in this study. (DOCX)
