# Supplementary figures and images for: High-level production and purification in a functional state of an extrasynaptic gamma-aminobutyric acid type A receptor containing α4β3δ subunits
Source: PLoS One. 2018 Jan 19;13(1):e0191583. doi: 10.1371/journal.pone.0191583 (PMC5774841; doi:10.1371/journal.pone.0191583)

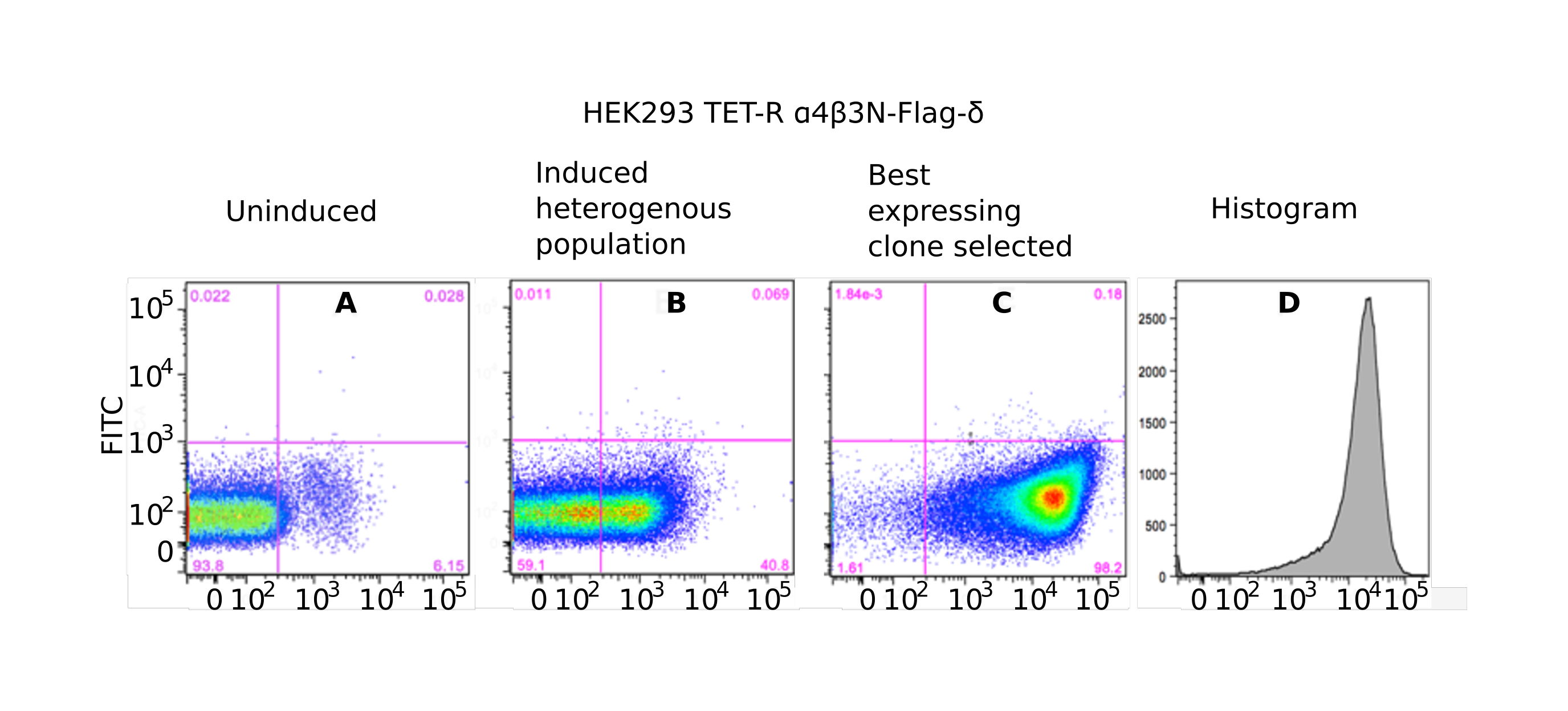

Supplement: S1 Fig — Panels A-D depict flow cytometry results at indicated stages of cell line development. (A) Transfected cells following antibiotic selection. (B) Cells as in panel A following 24 hours of tetracycline induction. (C) Monoclonal population of cells with the highest expression of N-Flag-δ used in the study, confirmed to have approximately 19 pmol/mg [3H]muscimol binding sites in the membrane fraction. (D) Histogram of cells in panel C. (TIF) [file pone.0191583.s001.tif]

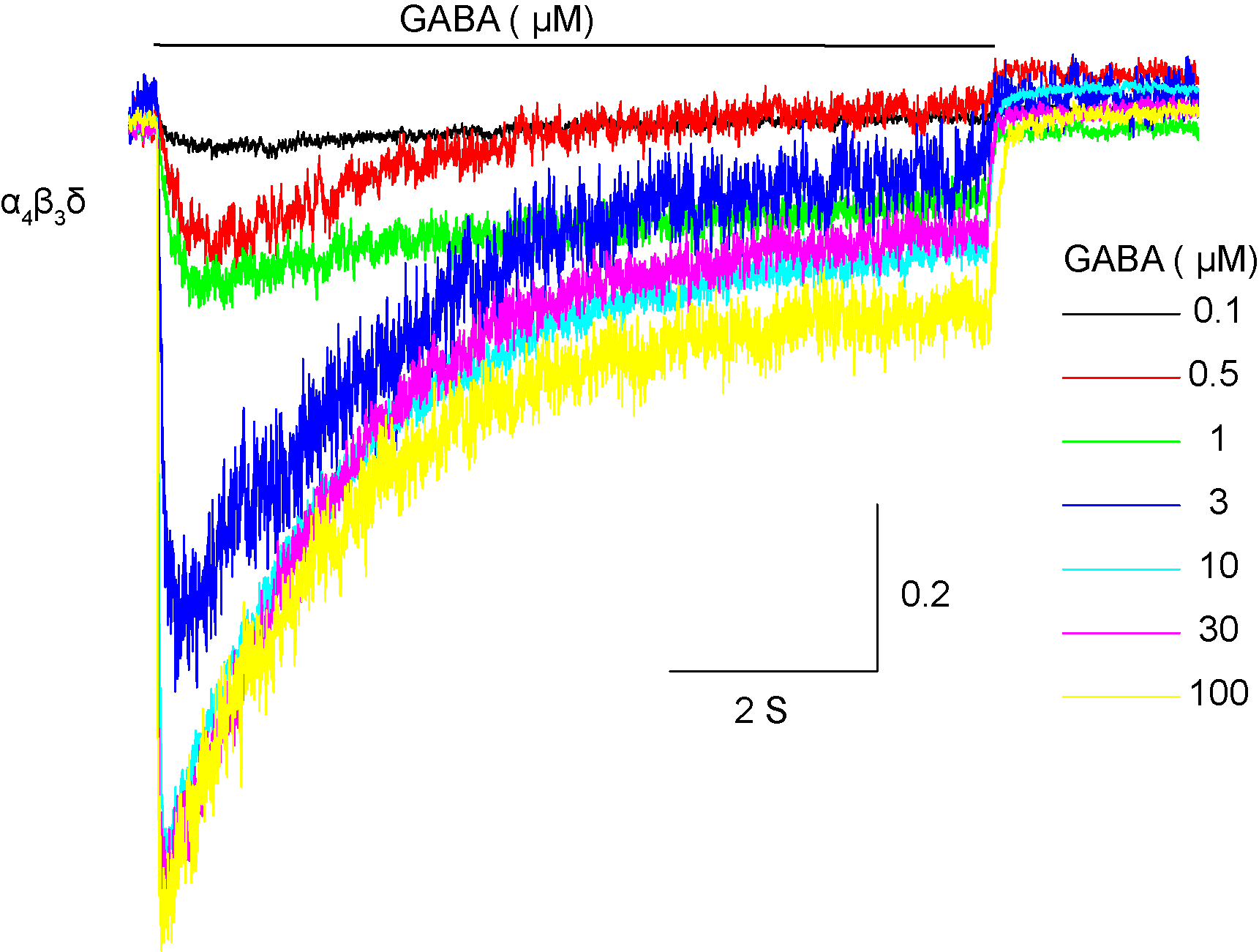

Supplement: S2 Fig — Currents were recorded using whole cell patch clamp technique from cells exposed to 0.1; 0.5; 1; 3; 10; 30; 100 μM of GABA for 8 seconds. Each concentration was recorded on 3–5 cells and peak amplitude was normalized to the recording obtained with 10 mM GABA on the same cell. Representative current for a single cell is shown. (TIF) [file pone.0191583.s002.tif]

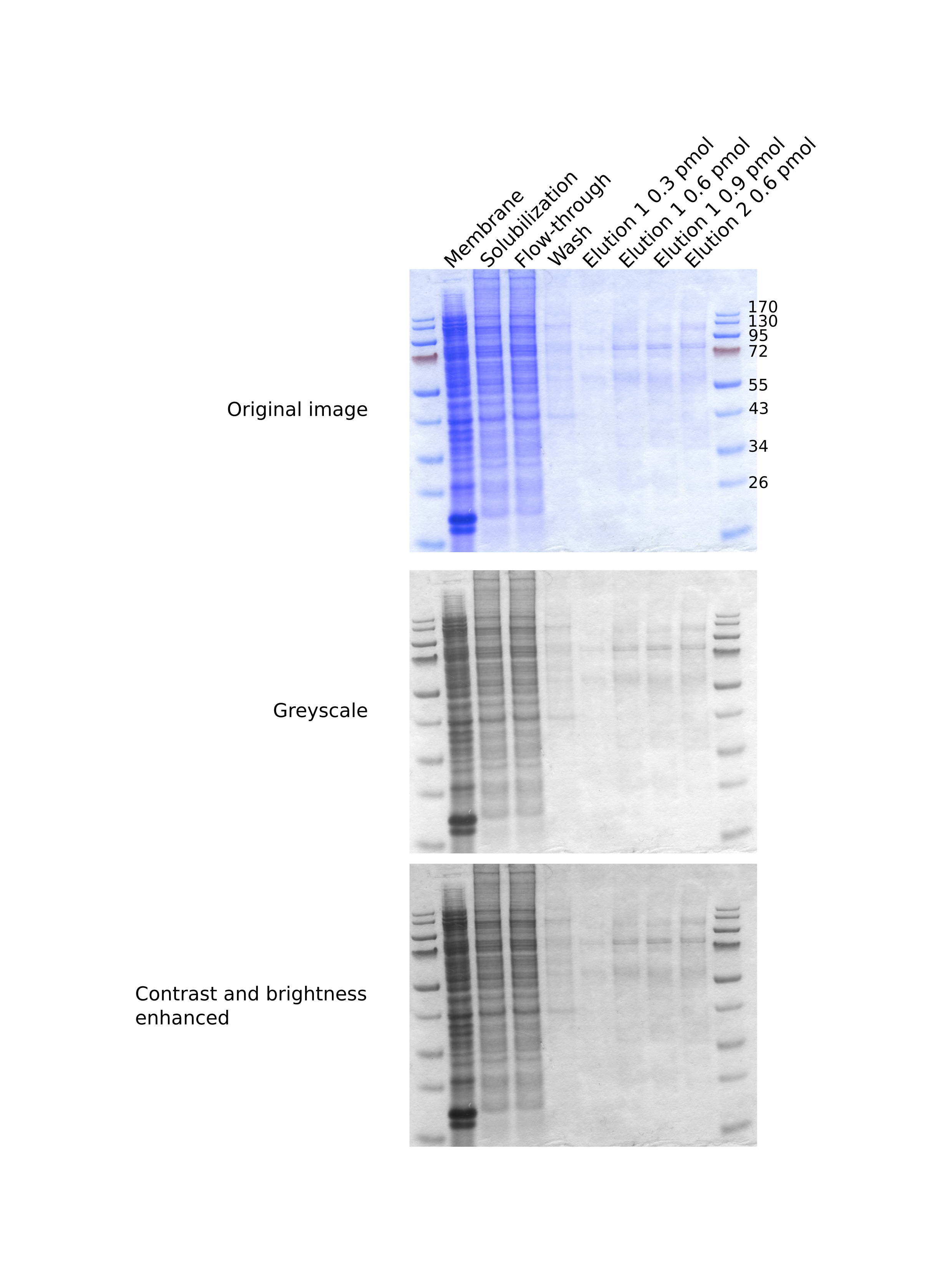

Supplement: S3 Fig — Samples collected throughout the purification procedure were resolved on 10% SDS-PAGE gel under denaturing conditions. Second lane (membrane) was loaded with 30 μg (0.86 pmol) of total membrane protein. Gel was subsequently fixed and stained with Coomassie Blue. A similar pattern was observed by Chiara and colleagues in photolabelling studies (Chiara et al., 2016). The main bands observed in the elution fractions represent individual subunits of the α4β3δ GABAA receptor. Numbers to the right represent molecular weights of the markers. Number of pmols in the Elution fractions loaded was determined by [3H]muscimol binding assay. Contrast and brightness of the bottom panel were adjusted uniformly to facilitate inspection. (TIF) [file pone.0191583.s003.tif]

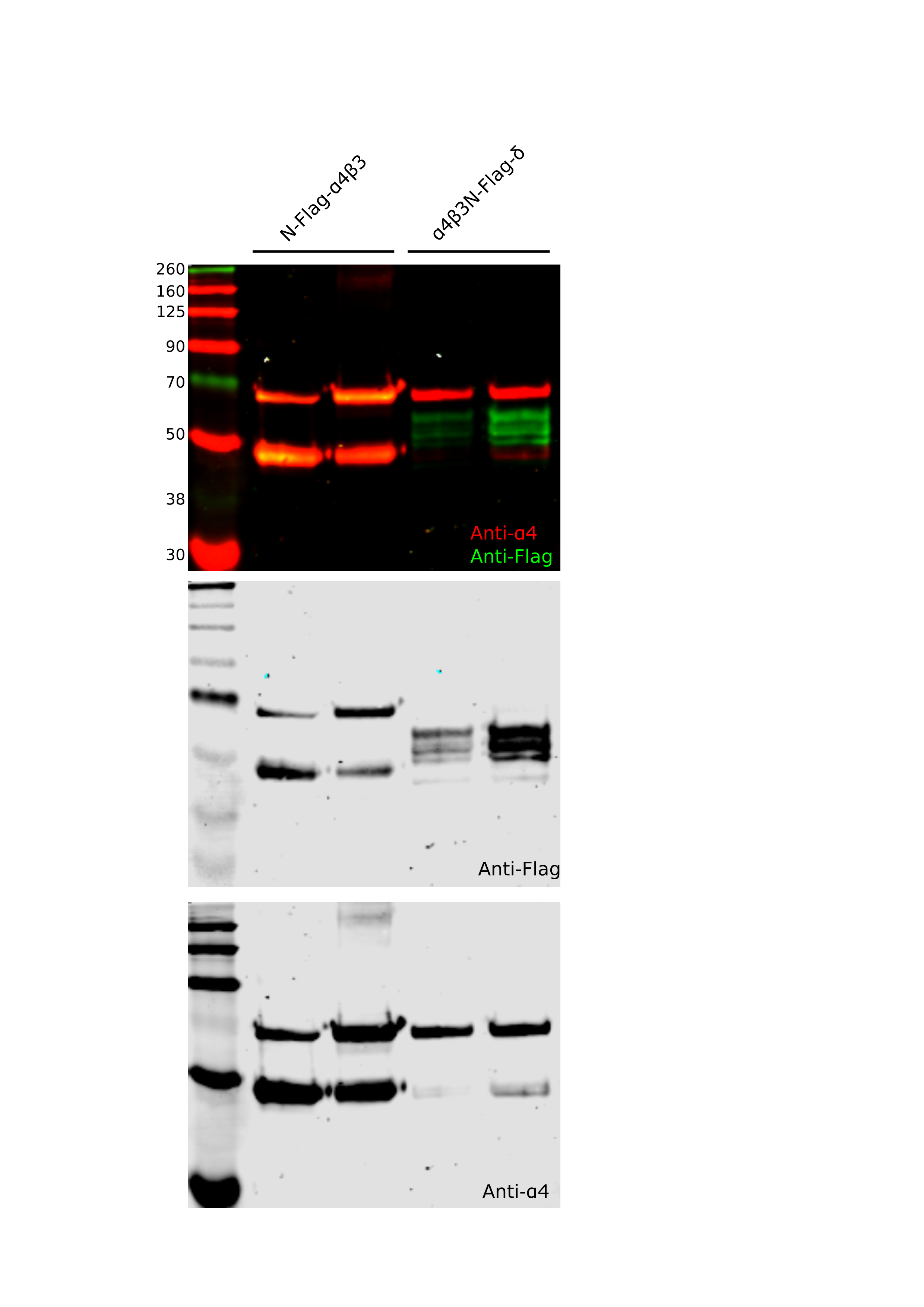

Supplement: S4 Fig — Whole immunoblot used to produce Fig 6A is presented. Two independent preparations for each reconstituted receptor is presented and those additional preparations flank the lanes shown in Fig 6A. Molecular weight standards are shown on the left hand side including their size in kDa. Lower two panels are greyscale representation of individual channels corresponding to immunoblot with antibodies as indicated. (TIF) [file pone.0191583.s004.tif]

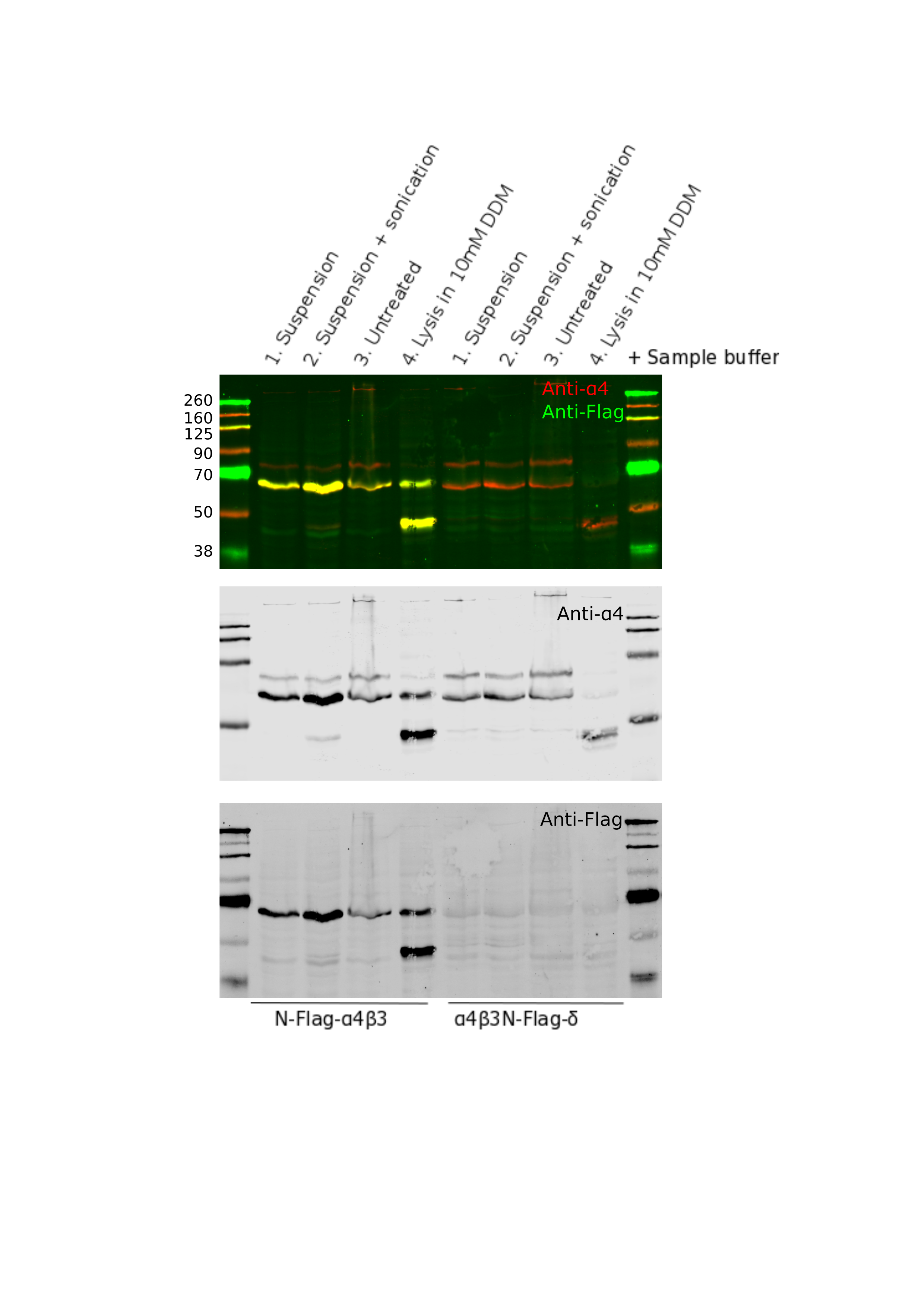

Supplement: S5 Fig — Whole immunoblot used to produce Fig 6B is presented. Immunoblot with polyclonal anti-α4-subunit antibody produced additional band above 70 kDa. This band was not observed on other occasions and it did not correlate with anti-Flag immunoblot on samples positive for N-Flag-α4β3, suggesting it to be non-specific. (TIF) [file pone.0191583.s005.tif]

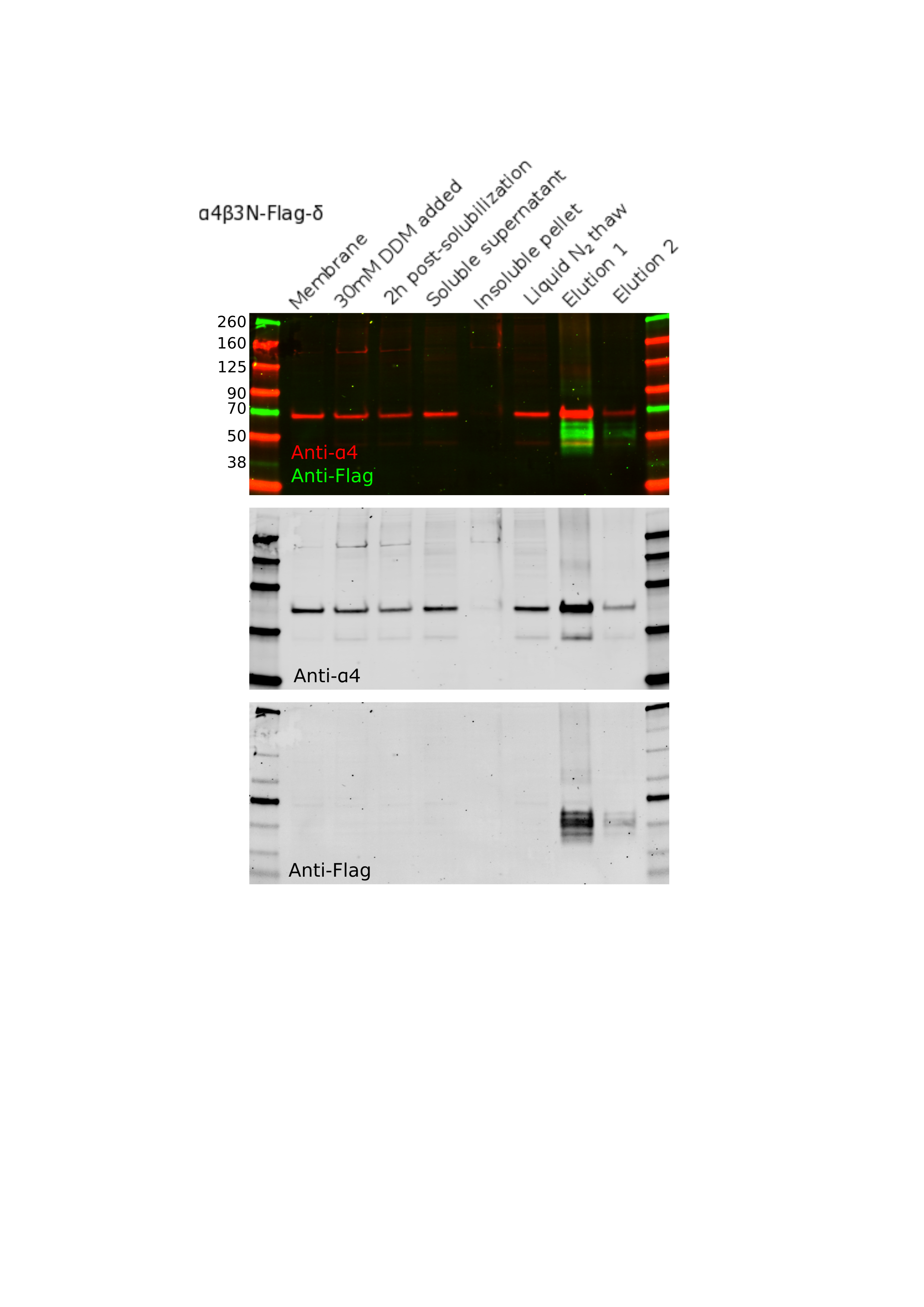

Supplement: S6 Fig — Whole immunoblot of α4β3N-Flag-δ receptor used to produce Fig 6C is presented. Molecular weight markers are flanking with their respective size in kDa indicated on the left hand side. (TIF) [file pone.0191583.s006.tif]

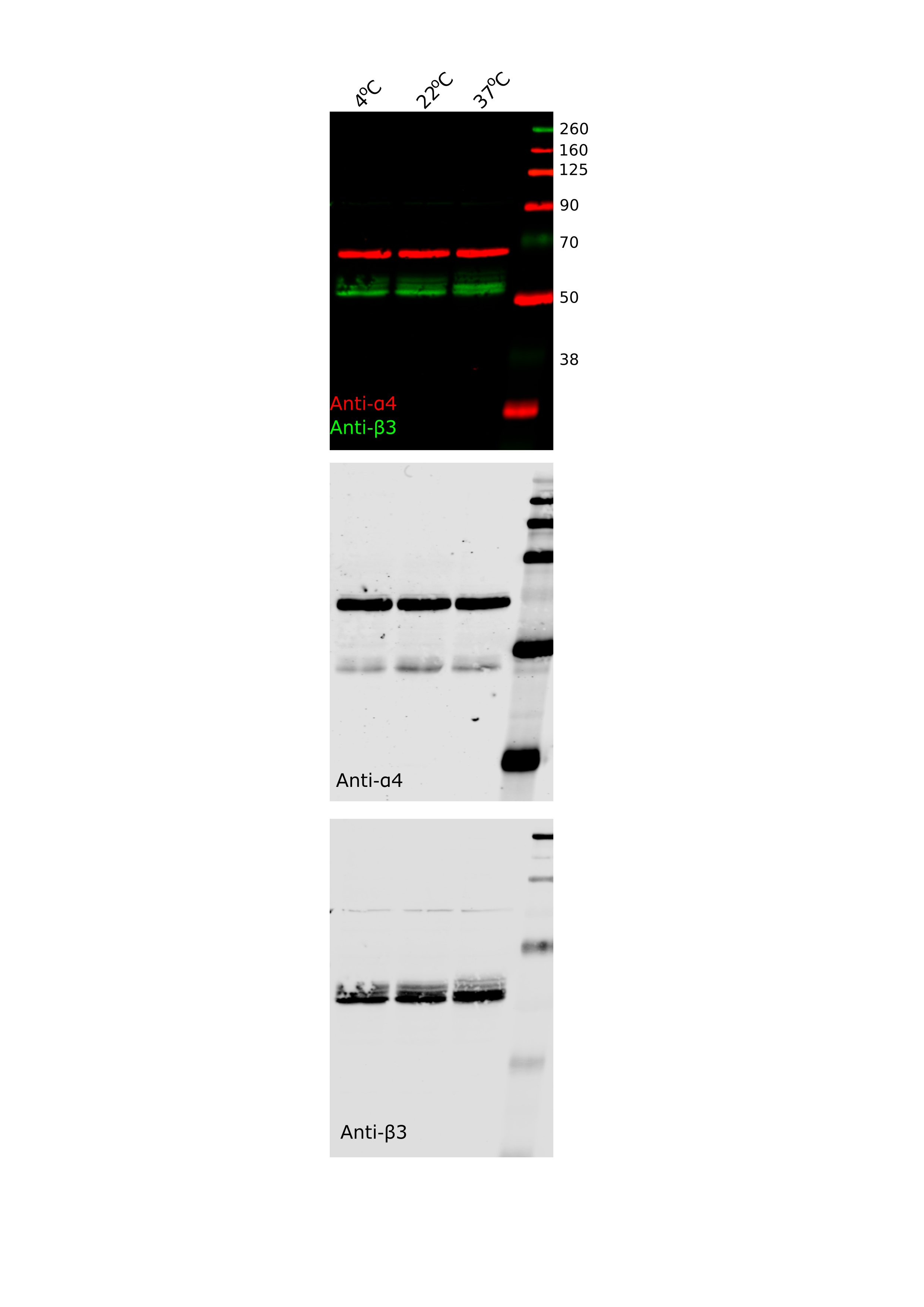

Supplement: S7 Fig — Whole immunoblot used to produce Fig 6D is presented. The molecular weight marker is shown on the left, with respective size indicated on the right hand side. The marker migrated at a slight angle. (TIF) [file pone.0191583.s007.tif]

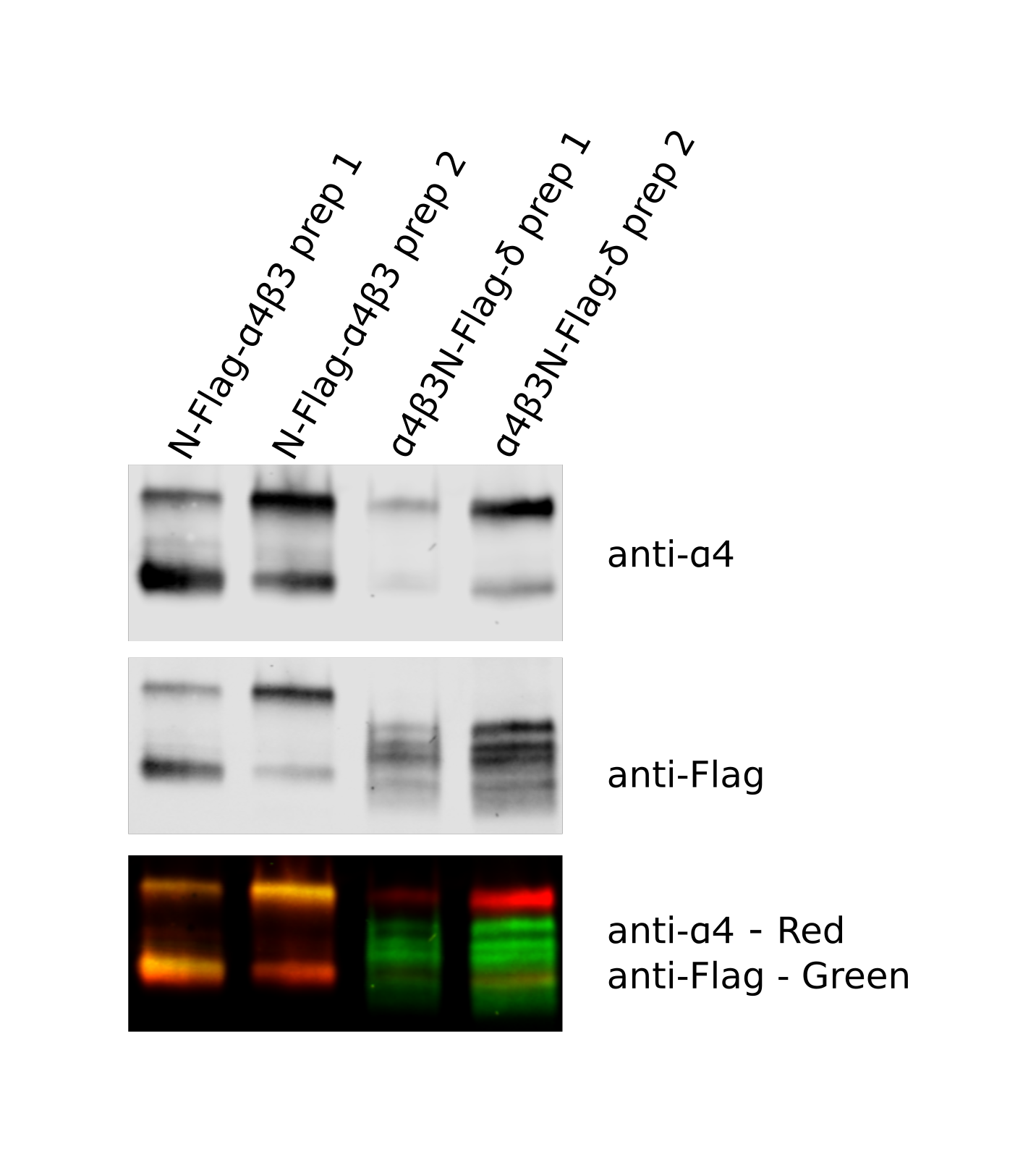

Supplement: S8 Fig — Reconstituted N-Flag-α4β3 and α4β3N-Flag-δ receptors from two independent purifications of each were analyzed by Western blotting. The bottom (color) panel represents the original membrane scan that was used for quantification. Grayscale panels are depicted to facilitate analysis. Immunoblots were uniformly adjusted for brightness and contrast. (TIF) [file pone.0191583.s008.tif]

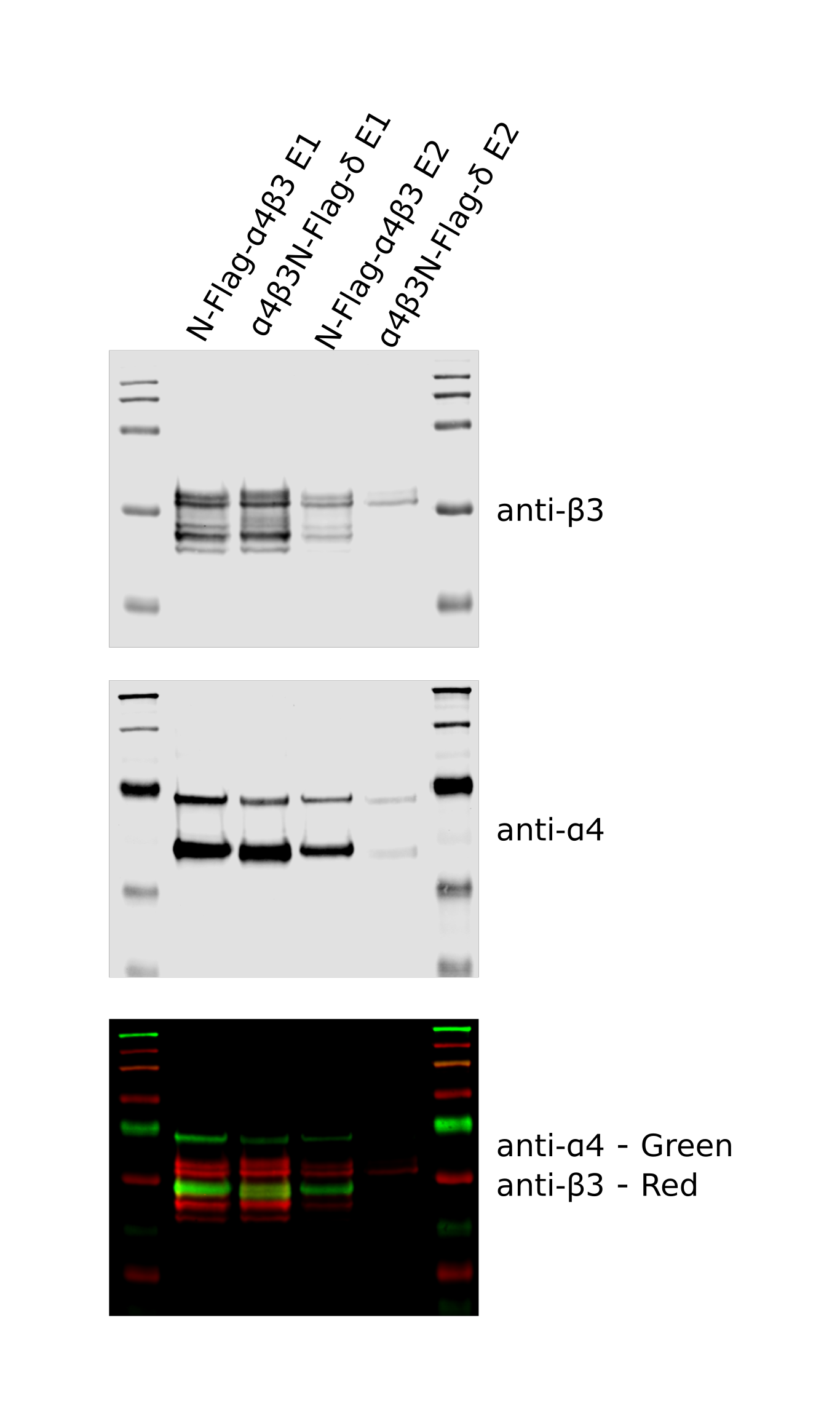

Supplement: S9 Fig — Elution 1 and elution 2 fractions of the reconstituted N-Flag-α4β3 and α4β3N-Flag-δ receptors were analyzed by Western blotting. The bottom (color) panel represents the original membrane scan that was used for quantification. Grayscale panels are depicted to facilitate analysis. Immunoblots were uniformly adjusted for brightness and contrast. (TIF) [file pone.0191583.s009.tif]
