# Supplementary material for: High-level production and purification in a functional state of an extrasynaptic gamma-aminobutyric acid type A receptor containing α4β3δ subunits
Source: PLoS One. 2018 Jan 19;13(1):e0191583. doi: 10.1371/journal.pone.0191583 (PMC5774841; doi:10.1371/journal.pone.0191583)
Supplement: S1 Table — (DOCX) [file pone.0191583.s010.docx]

**S1 Table. Primers used in the study**

| **Cloning primers** | |
| --- | --- |
| α4 - Forward | 5' AGATGGTTTCTGCCAAGAAGGTAC 3' |
| α4 - Reverse | 5' TTACATTAGACTTTCTGATTTCTCCATAGTGTC 3' |
| δ - Forward | 5' CATGGACGCGCCCGCCCGGCTG 3' |
| δ - Reverse | 5' GCTCACATGGCGTATGCCGCCCAG 3' |
|  |  |
| **Mutagenesis primers** | |
| N-Flag-δ - Forward | 5' GCGATGAATGACATCGGCGACTACAAAGACGATGACGACAAGGACTACGTGGGCTCC 3' |
| N-Flag-δ - Reverse | 5' GGAGCCCACGTAGTCCTTGTCGTCATCGTCTTTGTAGTCGCCGATGTCATTCATCGC 3' |
| N-Flag- α4 - Forward | 5' CTGGCGGTTTGTTTAAACGACTACAAAGACGATGACGACAAGGAATCCCCAGGACAGAAC 3' |
| N-Flag- α4 - Reverse | 5' GTTCTGTCCTGGGGATTCCTTGTCGTCATCGTCTTTGTAGTCGTTTAAACAAACCGCCAG 3' |
