# Supplementary material for: High-level production and purification in a functional state of an extrasynaptic gamma-aminobutyric acid type A receptor containing α4β3δ subunits
Source: PLoS One. 2018 Jan 19;13(1):e0191583. doi: 10.1371/journal.pone.0191583 (PMC5774841; doi:10.1371/journal.pone.0191583)
Supplement: S2 Table — (DOCX) [file pone.0191583.s011.docx]

**S2 Table. Antibodies used in the study**

| **Primary antibody** | **Manufacturer** | **Dilution Used** |
| --- | --- | --- |
| Anti-α4 rabbit polyclonal | Abcam, ab176274 | 1:1000 |
| Anti- β3 rabbit polyclonal | Novus Biologicals, NB300-199 | 1:250 |
| Anti- β3 mouse monoclonal | Sigma, SAB5200049 | 1:1000 |
| Anti-Flag mouse monoclonal | Sigma, F3165 | 1:2000 |
|  |  |  |
| **Secondary antibody** |  |  |
| IRDye 680RD anti-Mouse | Li-Cor, 926-6807 | 1:10,000 |
| IRDye 680RD anti-Rabbit | Li-Cor, 926-68073 | 1:10,000 |
| IRDye 800CW anti-Mouse | Li-Cor, 926-32212 | 1:10,000 |
| IRDye 800CW anti-Rabbit | Li-Cor, 926-3221 | 1:10,000 |
