# Supplementary material for: High-level production and purification in a functional state of an extrasynaptic gamma-aminobutyric acid type A receptor containing α4β3δ subunits
Source: PLoS One. 2018 Jan 19;13(1):e0191583. doi: 10.1371/journal.pone.0191583 (PMC5774841; doi:10.1371/journal.pone.0191583)
Supplement: S1 Text — (DOCX) [file pone.0191583.s012.docx]

## S1 Text. Subunit sequences used for preparation of genetic constructs

Human α4 based on Accession#NM_000809

Sequence used in N-Flag-α4β3

MVSAKKVPAIALSAGVSFALLRFLCLAVCLN***DYKDDDDK***ESPGQNQKEEKLCTENFTRILDSLLDGYDNRLRPGFGGPVTEVKTDIYVTSFGPVSDVEMEYTMDVFFRQTWIDKRLKYDGPIEILRLNNMMVTKVWTPDTFFRNGKKSVSHNMTAPNKLFRIMRNGTILYTMRLTISAECPMRLVDFPMDGHACPLKFGSYAYPKSEMIYTWTKGPEKSVEVPKESSSLVQYDLIGQTVSSETIKSITGEYIVMTVYFHLRRKMGYFMIQTYIPCIMTVILSQVSFWINKESVPARTVFGITTVLTMTTLSISARHSLPKVSYATAMDWFIAVCFAFVFSALIEFAAVNYFTNIQMEKAKRKTSKPPQEVPAAPVQREKHPEAPLQNTNANLNMRKRTNALVHSESDVGNRTEVGNHSSKSSTVVQESSKGTPRSYLASSPNPFSRANAAETISAARALPSASPTSIRTGYMPRKASVGSASTRHVFGSRLQRIKTTVNTIGATGKLSATPPPSAPPPSGSGTSKIDKYARILFPVTFGAFNMVYWVVYLSKDTMEKSESLM*

Sequence used in α4β3N-Flag- δ

MVSAKKVPAIALSAGVSFALLRFLCLAVCLNESPGQNQKEEKLCTENFTRILDSLLDGYDNRLRPGFGGPVTEVKTDIYVTSFGPVSDVEMEYTMDVFFRQTWIDKRLKYDGPIEILRLNNMMVTKVWTPDTFFRNGKKSVSHNMTAPNKLFRIMRNGTILYTMRLTISAECPMRLVDFPMDGHACPLKFGSYAYPKSEMIYTWTKGPEKSVEVPKESSSLVQYDLIGQTVSSETIKSITGEYIVMTVYFHLRRKMGYFMIQTYIPCIMTVILSQVSFWINKESVPARTVFGITTVLTMTTLSISARHSLPKVSYATAMDWFIAVCFAFVFSALIEFAAVNYFTNIQMEKAKRKTSKPPQEVPAAPVQREKHPEAPLQNTNANLNMRKRTNALVHSESDVGNRTEVGNHSSKSSTVVQESSKGTPRSYLASSPNPFSRANAAETISAARALPSASPTSIRTGYMPRKASVGSASTRHVFGSRLQRIKTTVNTIGATGKLSATPPPSAPPPSGSGTSKIDKYARILFPVTFGAFNMVYWVVYLSKDTMEKSESLM*

Human β3 wild type Accession NM_021912.3|

MCSGLLELLLPIWLSWTLGTRGSEPRSVNDPGNMSFVKETVDKLLKGYDIRLRPDFGGPPVCVGMNIDIASIDMVSEVNMDYTLTMYFQQYWRDKRLAYSGIPLNLTLDNRVADQLWVPDTYFLNDKKSFVHGVTVKNRMIRLHPDGTVLYGLRITTTAACMMDLRRYPLDEQNCTLEIESYGYTTDDIEFYWRGGDKAVTGVERIELPQFSIVEHRLVSRNVVFATGAYPRLSLSFRLKRNIGYFILQTYMPSILITILSWVSFWINYDASAARVALGITTVLTMTTINTHLRETLPKIPYVKAIDMYLMGCFVFVFLALLEYAFVNYIFFGRGPQRQKKLAEKTAKAKNDRSKSESNRVDAHGNILLTSLEVHNEMNEVSGGIGDTRNSAISFDNSGIQYRKQSMPREGHGRFLGDRSLPHKKTHLRRRSSQLKIKIPDLTDVNAIDRWSRIVFPFTFSLFNLVYWLYYVN*

Human N-Flag-δ based on (NM_000815)

MDAPARLLAPLLLLCAQQLRGTRAMNDIG***DYKDDDDK***DYVGSNLEISWLPNLDGLIAGYARNFRPGIGGPPVNVALALEVASIDHISEANMEYTMTVFLHQSWRDSRLSYNHTNETLGLDSRFVDKLWLPDTFIVNAKSAWFHDVTVENKLIRLQPDGVILYSIRITSTVACDMDLAKYPMDEQECMLDLESYGYSSEDIVYYWSESQEHIHGLDKLQLAQFTITSYRFTTELMNFKSAGQFPRLSLHFHLRRNRGVYIIQSYMPSVLLVAMSWVSFWISQAAVPARVSLGITTVLTMTTLMVSARSSLPRASAIKALDVYFWICYVFVFAALVEYAFAHFNADYRKKQKAKVKVSRPRAEMDVRNAIVLFSLSAAGVTQELAISRRQRRVPGNLMGSYRSVGVETGETKKEGAARSGGQGGIRARLRPIDADTIDIYARAVFPAAFAAVNVIYWAAYAM*

Underlined are signal peptide sequences predicted by the SignalP server (Petersen *et al*., 2011). In bold and italicized are Flag sequence inserted into α4- or δ-subunits.
